# Cheminformatics and artificial intelligence for accelerating agrochemical discovery

**DOI:** 10.3389/fchem.2023.1292027

**Published:** 2023-11-29

**Authors:** Yannick Djoumbou-Feunang, Jeremy Wilmot, John Kinney, Pritam Chanda, Pulan Yu, Avery Sader, Max Sharifi, Scott Smith, Junjun Ou, Jie Hu, Elizabeth Shipp, Dirk Tomandl, Siva P. Kumpatla

**Affiliations:** ^1^ Corteva Agriscience, Farming Solutions and Digital, Indianapolis, IN, United States; ^2^ Corteva Agriscience, Crop Protection Discovery and Development, Indianapolis, IN, United States; ^3^ Corteva Agriscience, Regulatory and Stewardship, Indianapolis, IN, United States; ^4^ Corteva Agriscience UK Limited, Regulation Innovation Center, Abingdon, United Kingdom; ^5^ Atomwise, San Francisco, CA, United States; ^6^ Karyosoft Inc, Carmel, IN, United States

**Keywords:** agrochemicals, DMTA cycle, cheminformatics, artificial intelligence, machine learning, lead generation, lead optimization, sustainability

## Abstract

The global cost-benefit analysis of pesticide use during the last 30 years has been characterized by a significant increase during the period from 1990 to 2007 followed by a decline. This observation can be attributed to several factors including, but not limited to, pest resistance, lack of novelty with respect to modes of action or classes of chemistry, and regulatory action. Due to current and projected increases of the global population, it is evident that the demand for food, and consequently, the usage of pesticides to improve yields will increase. Addressing these challenges and needs while promoting new crop protection agents through an increasingly stringent regulatory landscape requires the development and integration of infrastructures for innovative, cost- and time-effective discovery and development of novel and sustainable molecules. Significant advances in artificial intelligence (AI) and cheminformatics over the last two decades have improved the decision-making power of research scientists in the discovery of bioactive molecules. AI- and cheminformatics-driven molecule discovery offers the opportunity of moving experiments from the greenhouse to a virtual environment where thousands to billions of molecules can be investigated at a rapid pace, providing unbiased hypothesis for lead generation, optimization, and effective suggestions for compound synthesis and testing. To date, this is illustrated to a far lesser extent in the publicly available agrochemical research literature compared to drug discovery. In this review, we provide an overview of the crop protection discovery pipeline and how traditional, cheminformatics, and AI technologies can help to address the needs and challenges of agrochemical discovery towards rapidly developing novel and more sustainable products.

## 1 Introduction

The development and application of computational tools has accelerated the pace of research and product development in diverse domains. Considering the impact computation has created, it was no exaggeration when it was stated that ‘behind every great scientific finding in the modern age, from astronomy to zoology, there is a computer’ (Perkel, 2021). Following decades of impressive growth, both pharmaceutical and agricultural industries have faced several challenges in bringing new products to market. Elevated costs (W. Zhang, 2018), increased regulatory requirements, and the need for differentiated products with novel modes of action (Sparks et al., 2018) are requiring unprecedented research and development investments to account for attrition in the pipeline and success in developing promising products (McDougall, 2016; Wouters et al., 2020).

Agrochemical product development, while having some parallels to pharmaceutical industry, has its own set of challenges that include addressing resistance development in pests (Siegwart et al., 2015; Hawkins et al., 2019), identifying sustainable chemistries (Whiteker, 2019), striking a balance with available genetically modified solutions, and competing with alternative and emerging technologies (Nishimoto, 2019). The data explosion and significant developments in data analytics that occurred throughout recent decades have provided means to address these challenges. In fact, this has further motivated the creation of newer, faster, and more scalable computational methods and tools for data generation, analysis, and hypothesis generation with the potential of decreasing the cost and time requirements for research and development of bioactive molecules.

Cheminformatics, also referred to as chemoinformatics, is the application of computer and informatics technologies to chemistry and has revolutionized the understanding of chemistry by improving the speed of development of novel products (Engel, 2006). It is a multidisciplinary field that employs tools and learnings from chemistry, biology, biochemistry, mathematics, statistics, and a host of other fields. Although the specific term cheminformatics has been in circulation for a little over two decades, its foundations can be traced back to the middle of last century when the conversion of chemical literature and mass spectra from print to electronic formats was initiated, database search systems were developed, and the widely used substructure matching algorithm came into existence (W. L. Chen, 2006; Ray and Kirsch, 1957). These seminal advances were followed by notable progress in subsequent decades that includes the development of chemical database retrieval systems and AI-based expert systems in the 1960s, creation of major chemical databases and development of binary fingerprints for substructure and similarity searches in the 1980s, introduction of new structural representation formats such as the Simplified Molecular-Input-Line-Entry System (SMILES) in the 1980s (Weininger, 1988) and the IUPAC International Chemical Identifier in the 2000s (Goodman et al., 2021), and the development of the first Machine Learning (ML) models to predict activity and physical properties in the 1990s (W. L. Chen, 2006). A key aspect of computational modeling that became a vital part of modern cheminformatics was the correlation of molecular structures with their biological function, which came to be known as Quantitative Structure-Activity Relationship (QSAR) (W. L. Chen, 2006). While linear models, partial least squares (PLS), and related traditional mathematical techniques enabled initial successes in QSAR modeling, the use of artificial neural networks (ANNs) for QSAR studies, first reported in 1990, was in prominence for several years until the onset of Random Forest and Support Vector Machine (SVM) approaches (Aoyama et al., 1990). The revolutionary successes of deep neural network (DNN) architectures in imaging (Baskin et al., 2016) brought about a renaissance of neural network architectures in a host of new and emerging tools for almost all steps in the discovery and development pipeline in both pharma and agricultural sectors since the mid-2010s. These include transformer-based ANNs for accurate conversion of chemical notations and the prediction of physicochemical properties, generative adversarial networks (GANs) for exploring chemical space as well as optimizing the functionality of known compounds, and deep learning (DL) and generative methods for intelligent navigation of small molecule space (Lo et al., 2019; Kell et al., 2020; Blanchard et al., 2021; Krasnov et al., 2021).

Several excellent reviews exist that describe the role of cheminformatics in drug discovery (Begam and Satheesh Kumar 2012; Lo et al., 2018; Lo et al., 2019; Chen and Kirchmair, 2020; Martinez-Mayorga et al., 2020). The motivation behind this article is to provide such a review for agrochemical discovery and development and to highlight how cheminformatics and AI tools are impacting the efficiency and speed of this process and in realizing the goals of developing sustainable and environmentally friendly products.

## 2 The crop protection discovery pipeline

**FIGURE 1:**
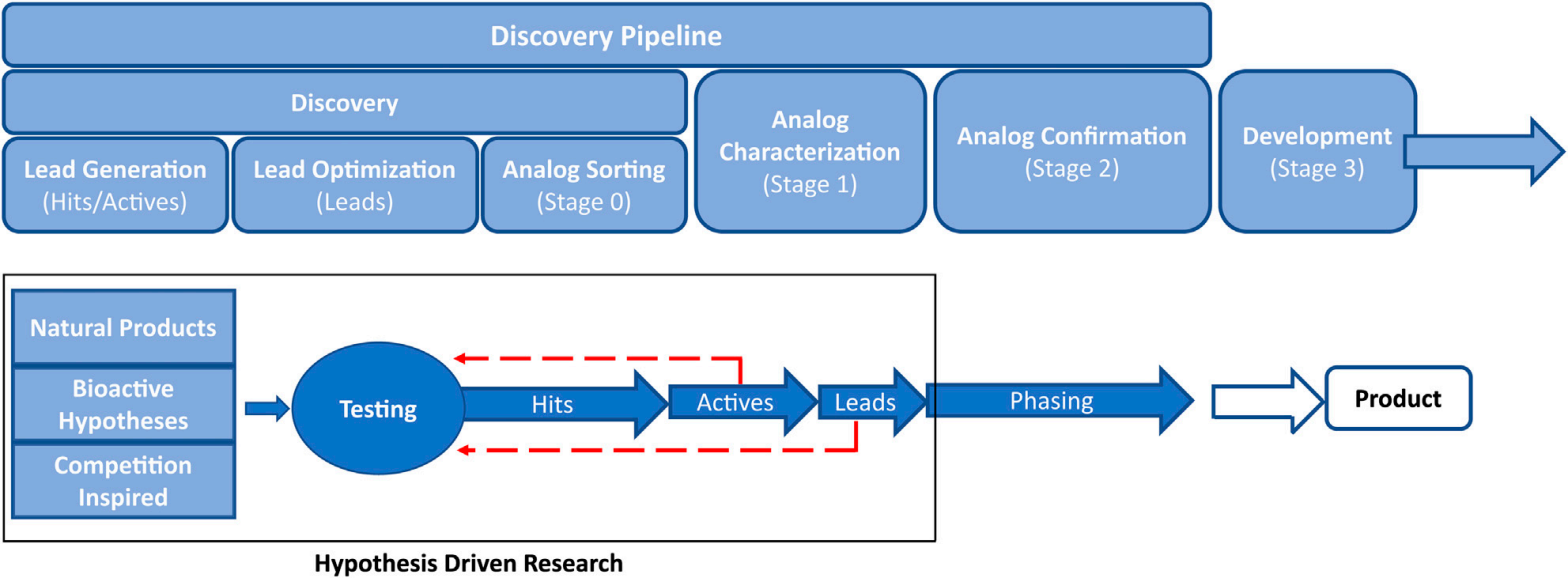
Overview of the crop protection discovery pipeline.

The value of cheminformatics has been demonstrated in all stages of the pipeline used for the discovery of new crop protection active ingredients. In this review, we will refer to the crop protection pipeline (See Figure 1) as hit → active → lead generation → lead optimization as outlined by Loso *et al.* (Loso et al., 2017). Briefly, a ‘hit’ is defined as a compound that passes an activity threshold in the earliest tests (typically high-throughput screening) while an ‘active’ is a synthetically actionable compound with activity against target species that makes it a reasonable starting point for further exploration. A ‘lead’ molecule has an activity profile and novelty that warrant significant investment. Each stage of the pipeline has unique challenges that have the potential to be partially or entirely addressed with cheminformatics technology.

There are many approaches to begin the search for new hits to feed into the pipeline (Lamberth et al., 2013). Some examples include retrospective searches through databases for hints of activity from historical assays, known target site binders from the pharma literature, genome searches for cross-species target sites, pesticidal natural products (Lorsbach et al., 2019; Meyer et al., 2021; Sparks et al., 2021), novel fragments (Zhu et al., 2011), and competition inspired hit generation (Lahm et al., 2007). The success of any of these approaches hinges on the ability to quickly and accurately search across multiple chemical structure databases of millions of structures (company databases and literature) to billions or more in the case of virtual databases such as Enamine’s REAL offerings (Grygorenko et al., 2020). These search results should be easily narrowed down to those compounds that are predicted to not only have activity against target pests, but also have ag-like physicochemical properties (Zhang et al., 2018). It is also desirable to limit screening decks to diverse but relevant subsets, that ideally are small so they can be built upon in subsequent optimizations.

Once a hit has been identified, the advancement to an active typically involves broad exploration of nearby chemical space, Structure-Activity Relationship (SAR) exploration, and scaffold hops with testing to define the general areas of activity (e.g.: lepidopterans vs. coleopterans, broadleaf vs. grasses, ascomycetes vs. basidiomycetes, etc.). Compound sourcing at this stage is similar to hit generation such that compounds available within the company compound library, from commercial vendors, and from direct synthesis are all utilized. Broadly trained predictive models are generally still relevant, as the chemical space in which active generation takes place is still large and there are insufficient data points to create a meaningful active-specific predictive model.

At the active-to-lead stage of the pipeline, the SAR exploration becomes narrower, however, there is still significant probing of available chemical space. Typically, from this point forward all additional molecules are custom prepared, as commercial vendor chemical space is exhausted. It is at this point that there are usually enough molecules tested to generate area-specific predictive models. Physical property guidance becomes even more important. Initial work on target site identification begins, if it is not already known. The general pest spectrum is characterized, and a potential product concept is sketched out.

Once an active is advanced to lead, it is then considered a full-fledged project with significant resources made available. The SAR has been narrowed to the point that each portion of the molecule is deep-drilled due to the need for optimization of many parameters simultaneously (potency, selectivity, toxicity, environmental fate, cost of manufacture, etc.). Target site and mode of action confirmation become imperative, which can then further inform models.

## 3 Cheminformatics and AI for the design-make-test-analyze cycle

**FIGURE 2:**
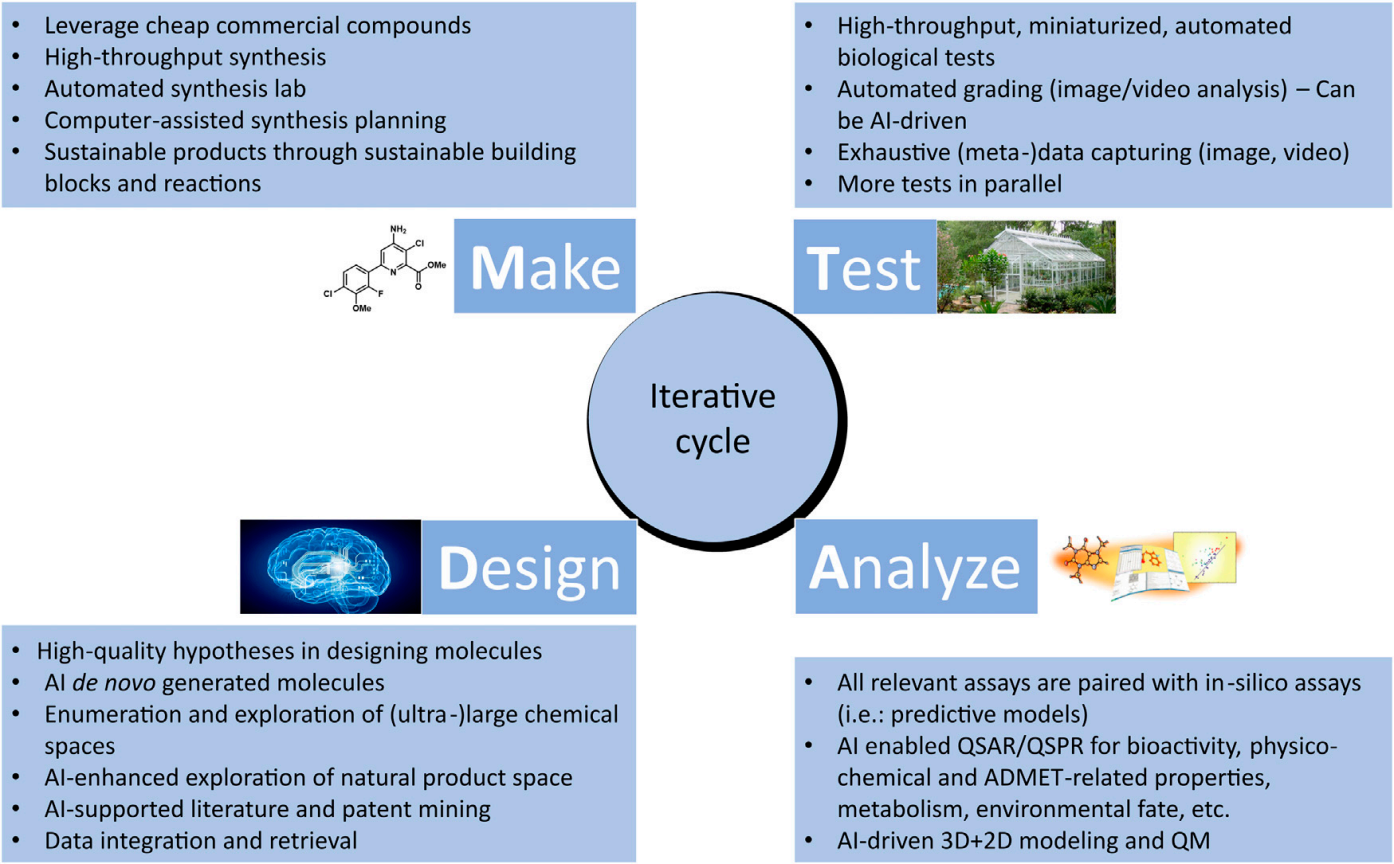
Overview of the iterative Design-Make-Test-Analyze (DMTA) cycle in agrochemical discovery. Cheminformatics and artificial intelligence play an increasingly significant role in each of the phases.

The Design-Make-Test-Analyze (DMTA) cycle is a central, iterative process consisting of interdependent steps that aim at efficiently designing, testing, and validating hypotheses, upon which data generated through experiments are analyzed, in order to discover new information that advances the discovery and optimization of leads (Plowright et al., 2012) (See Figure 2). Through several cycles, chemical hits are gradually optimized with respect to activity, selectivity, toxicity, and stability, into actives and eventually into more efficient lead molecules. Subsequently, selected lead molecules are further assessed using advanced models before development is initiated (Andersson et al., 2009). In this section, we will discuss how cheminformatics and AI are enhancing the pace and efficiency of DMTA cycle. In addition, we will highlight some of the challenges that need to be addressed to further accelerate the digitalization and automation as well as improve the success rate of DMTA processes in the discovery and optimization of sustainable agrochemicals.

### 3.1 Design

**FIGURE 3:**
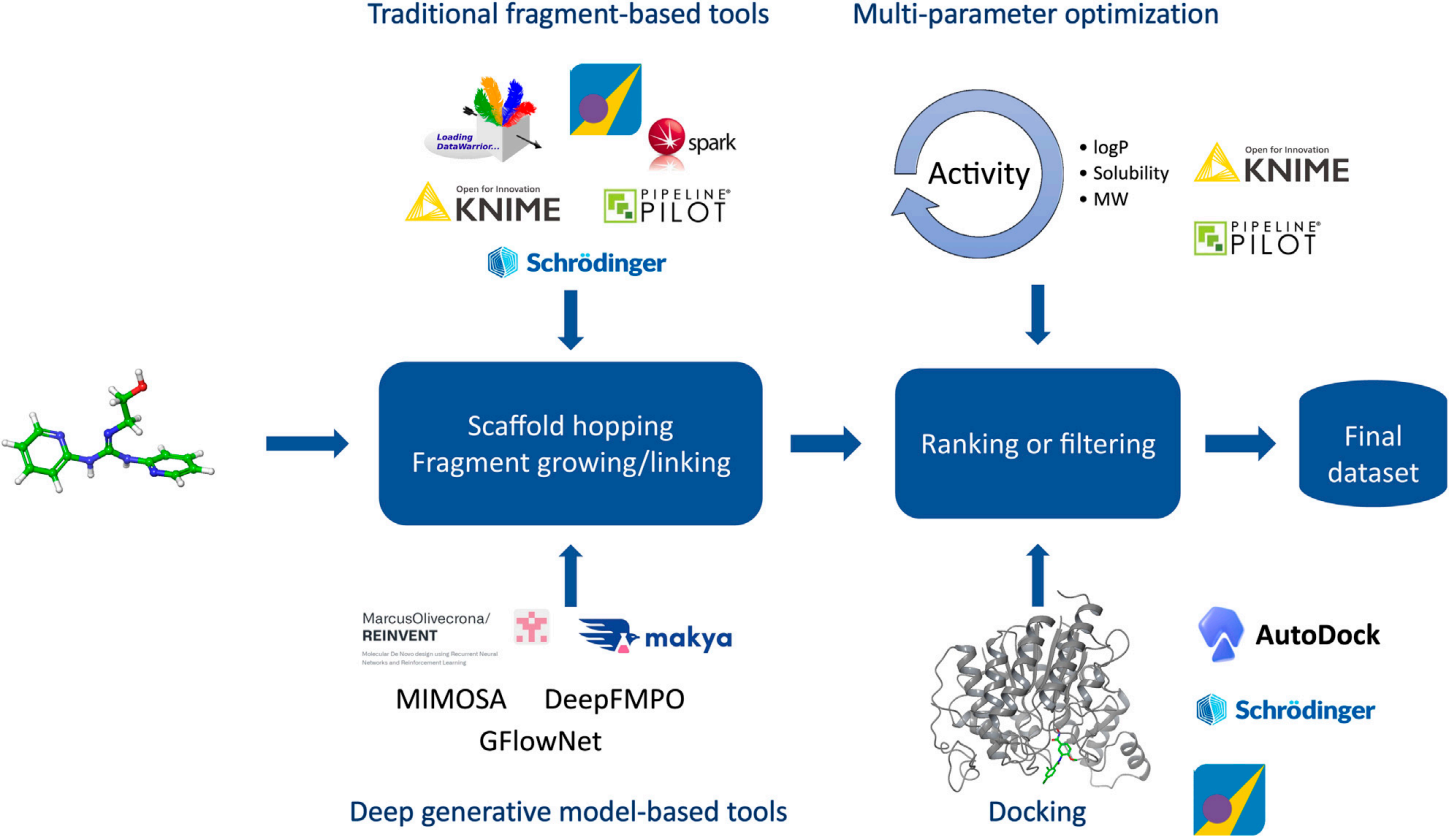
Overview of the molecular design workflow. Some examples of computational tools used in the design phase are provided.

Molecular design closely ties to the design cycle of the widely accepted concept of iterative lead discovery. Its primary goal is to deliver new chemical entities with specified properties and potencies (Kuhn et al., 2016); however, those properties (e.g.: physicochemical, ADME-Tox properties) can vary greatly from the pharmaceutical industry (Tice, 2001). Molecular design includes two critical steps - generating a pool of candidates, and using molecular scoring strategies to select molecules from the collection for different disciplines in the agrochemical (agchem) industry, such as insect management, weed management (Gandy et al., 2015; Quareshy et al., 2018), and crop disease management, each of which has differing physiochemical property requirements (Avram et al., 2014; Zhang et al., 2018) (See Figure 3). At the hit generation stage, molecules with appropriate physical properties should be chosen since targets tend to gain mass during the active and lead generation process within the bounds of a given discipline. Since this stage also contains the largest possible chemical space, tools that accurately predict these properties quickly and display the results to a user alongside relevant activity-based metrics (e.g.: predicted assay activity, similarity to a query structure with known activity, relative location in known chemical space, etc.) in an intuitive and responsive manner are particularly important. Rapid searching and virtual screening of billions of compounds in modern commercial screening collections can be accomplished using tools such as fastROCS (OpenEye Scientific, 2023a), Ftrees (BioSolveIT, 2023a), and InfiniSee (BioSolveIT, 2023b) (See Table 1). The optimization of targets from hits to actives and leads should adhere as closely as possible to principles of green chemistry, namely low use rates, low ecological toxicity, minimal bioaccumulation, and thorough breakdown into benign fragments (Casida, 2012; Whiteker, 2019). Therefore, computational methods used at this stage such as QSAR and traditional ML and deep learning (DL) largely focus on molecule generation, docking, virtual screens, or molecular properties prediction, with molecule generation being a popular application of cheminformatics capability.

**TABLE 1:** Examples of software tools used in molecular design.

Name	Description	References/Examples
RDKit	Cheminformatics	RDKit, (2023)
MOE	Molecular design, cheminformatics, QM, MM, QSAR, MD	ULC, C.C.G. (2023)
Data Warrior	Molecular design, cheminformatics	Sander et al. (2015)
KNIME	Data analysis, visualization, machine learning, deep learning, workflow	Berthold et al. (2008)
Pipeline Pilot	Data analysis, visualization, machine learning, deep learning, workflow	Dassault Systèmes SE, (2023)
Maestro	Molecular design, cheminformatics, QM, MM, QSAR, MD	Schrödinger, (2023b)
Spark	Molecular design	Cresset, (2023)
DeepFMPO	Deep learning, generative *de novo* design, reinforcement learning	Ståhl et al. (2019)
REINVENT	Deep learning, generative *de novo* design, multi-parametric optimization, reinforcement learning	Blaschke, Arús-Pous et al. (2020)
Makya	Deep learning, generative *de novo* design, multi-parametric optimization	Iktos, (2023a)
LillyMol	Cheminformatics	Eli Lilly & Co, (2019)
FastROCS™	Virtual Screening, Lead Hopping & Shape Clustering	OpenEye Scientific (2023b)
Ftrees	Virtual Screening	BioSolveIT, (2023a)
InfiniSee	Virtual Screening	BioSolveIT, (2023b)
Others	Deep learning, generative *de novo* design, multi-parametric optimization	Ståhl et al. (2019); Chuang et al. (2020), Khemchandani et al. (2020); Grechishnikova, (2021); Krishnan et al. (2022)

Historically, molecule generation included creating novel molecules from scratch and modifying structures based on scaffolds or fragments with demonstrated activity by bioisostere replacement, scaffold hopping/replacement, attaching functional groups, or linking multiple fragments. These functions have been implemented in popular tools such as DataWarrior (Sander et al., 2015), and KNIME (Berthold et al., 2008) (See Table 1). Early efforts in this area mostly prioritized the development of heuristic algorithms that focused on molecules predicted to be highly active and with desired properties (Sliwoski et al., 2013). The accumulation of data and advancement of ML methods are replacing these heuristics with evolving DL methods (Mater and Coote, 2019; Paul et al., 2021). Deep generative models (DGMs), leveraging the power of DNN architecture, are designed to learn latent representations of molecules even within a low-data setting and have a function to approximate the true distribution from which new compounds with desired molecular properties are sampled (Michael A. et al., 2021). Based on the architecture, these models can be categorized into (variational and adversarial) autoencoders (Blaschke et al., 2018; Richards and Groener, 2022), generative adversarial networks (GANs) (Méndez-Lucio et al., 2020; Abbasi et al., 2022), recurrent neural networks (RNN) with long short-term memory (LSTM) and gated recurrent unit (GRU) variants (Segler et al., 2018; He et al., 2021), and hybrid models combining deep generative models with reinforcement learning (RL) (Elton et al., 2019; Xue et al., 2019; Pereira et al., 2021) or autoencoders (Prykhodko et al., 2019). RL (Ståhl et al., 2019; Blaschke et al., 2020; Langevin et al., 2020) or conditional generative models (Kang and Cho, 2019; Sagar, 2020) speed up the process by generating only the molecules with desired properties or interesting scaffolds. Most DGMs take SMILES strings as inputs and then use a Variational Autoencoder (VAE) with Bayesian optimization in the latent space to generate molecules. Instead of generating molecules atom by atom, fragment-based language models can significantly reduce chemically invalid or duplicate compounds (Podda et al., 2020) as well as achieve comparable performance with fewer parameters and less training data (Chen et al., 2020). To further reduce the rate of chemically invalid generated molecules, Krenn *et al.* (Krenn et al., 2020) have introduced SELF-referencIng Embedded Strings (SELFIES), a more robust string-based representation of molecules. They demonstrated that VAEs and GANs using SELFIES generated only chemically valid molecules. Moreover, the generated sets of molecules were orders of magnitude more diverse when using SELFIES compared to SMILES strings. SELFIES were implemented in PASITHEA (Shen et al., 2021), a deep generative tool that applies “inceptionism” to propose new molecules with desired properties. Other popular approaches, such as Graph Neural Network (GNNs) have also been used in the generation of molecules (Shi et al., 2020; Mercado et al., 2021). GNNs, such as graph convolutional networks (GCNs) or message passing neural networks (MPNN), take graph-structured data as input and output a latent representation for the input graph. To improve performance, DGMs can be combined with each other (Méndez-Lucio et al., 2020) or other traditional ML algorithms (Blanchard et al., 2021). Metrics such as speed, coverage of chemical space, novelty, diversity, Kullback–Leibler (KL) divergence, and Fréchet ChemNet distance (Brown et al., 2019; Polykovskiy et al., 2020; Jie et al., 2021), among others, are widely used to evaluate their performance. The resulting molecules are then screened by agchem-related physiochemical property filters or pesticide-likeness scores (Zhang et al., 2018), predictive models trained by machine learning methods (Ray et al., 2017), or docking with protein models or homology models (Durrant et al., 2009; Chevillard et al., 2018; Hefke et al., 2020). In contrast to drug-likeness scores such as Quantitative Estimate of Drug likeness (QED) or drug-likeness models, the pesticide-likeness scores or models should include not only parameters related to bioactivity but also environmental effects such as volatilization, wash-off, photolysis, ecological toxicity, bioaccumulation, and soil metabolism for sustainability as well as the biodiversity of pests and usage conditions (Avram et al., 2014; Ouyang et al., 2021). The use of DGMs for molecule generation (Fromer and Coley, 2022) is promising but the challenges remain in how to improve diversity, novelty, and synthesizability (Benhenda, 2017; Gao and Coley, 2020), among other factors, within a multiparameter optimization framework. The advent of DGM has provided the opportunity to significantly improve the automated generation of molecules with desired properties and/or scaffolds using tools such as REINVENT and COMA, and thus, accelerate the advancement of molecules throughout the pipeline (Arús-Pous et al., 2020; Blaschke et al., 2020; Choi et al., 2023). The use of models trained on domain-relevant data, including the generative model, and associated scoring functions (e.g.: pharmacophore scoring) can lead to higher discovery rates of actionable and synthesizable compounds. For instance, by integrating pharmacophore features (e.g.: aromaticity, hydrophobicity) into the training of a REINVENT agent network, Yoshimori *et al.* (Yoshimori et al., 2021) were able synthesize nine DDR1 inhibitors with nanomolar potency. Moreover, recent works have introduced deep learning-based, protein-target driven *de novo* design approaches where the generative model takes protein specific information (e.g.: primary structure) to generate candidate ligands optimized towards various parameters (e.g.: high binding affinity, low toxicity) (Born et al., 2021; Zhang et al., 2023). While the methods used different generative algorithms and representations, they were able to propose ligands to relevant protein targets. Overall, the success of *de novo* generative design projects requires that goals be clearly defined by discovery teams, and priority be put on sampling strategy and efficiency, as illustrated in a benchmarking study by Gao *et al.* (Gao et al., 2022).

In contrast to the approach of creating novel molecules or modifying existing scaffolds and fragments, it is often desirable to screen libraries of compounds for novel hits. Molecular docking is a structure-based method that uses a search algorithm to generate ligand binding poses and a scoring function to quantitatively rank them. A common pitfall lies in the generation of false positives during ranking, either by failure to predict the correct pose of true ligands or by failure to discern between true ligands and decoys (Warren et al., 2006). Machine learning methods have shown promise in addressing these issues. For example, a support vector machine (SVM) regression analysis was used to score targets of AKT serine/threonine kinase 1, which led to the discovery of nanomolar inhibitors not attained with classical scoring functions (Zhan et al., 2014). Convolutional Neural Networks (CNN) algorithms have shown success at improving binding pose prediction by extracting features from protein-ligand complexes by analyzing their three-dimensional images (Ragoza et al., 2017). Incorporating machine learning into docking protocols is not without its share of issues. Neither protein-ligand structures nor sufficient data to develop a training set are guaranteed in agrochemical discovery. Moreover, the use of DL algorithms has been shown to fail compared to standard docking protocols in some cases (Gentile et al., 2020). As an alternative to developing novel scoring functions, Jimenéz-Luna *et al.* employed DL to rationally choose between standard docking protocols for a given protein-ligand pair with modest success (Jiménez-Luna et al., 2020a). Machine learning methods applied to docking and structure-based virtual screening are in a constant state of improvement, however, their utility in agrochemical discovery remains to be proven.

### 3.2 Make

The synthesis of chemical compounds is executed during the lead optimization and regulatory assessment phases, as well as once the final product is ready for commercialization. Hundreds of ideas and hypotheses can be generated in a relatively cost- and time-efficient manner during the design phase; however, the capability to convert these ideas into real and testable compounds remains one of the bottlenecks in the discovery process (Andersson et al., 2009). Because of the substantial number of assays to be run on target species as well as non-target species such as crops, much greater quantities of compounds are generally required compared to pharmaceutical research. It is thus critical that the synthesis of compounds is efficient, especially once an active has been optimized into a lead molecule. Generally, the synthesis of molecules involves: 1) selection of efficient synthetic routes for target compounds; 2) acquisition of building blocks and reagents; and 3) execution of the synthesis and purification phases. Cheminformatics and AI tools can be used in each of these phases to accelerate the process and reduce failures in the making of the novel molecules (Venkatasubramanian and Mann, 2022).

The decision on how to synthesize a novel compound is not only essential within the DMTA cycle, but also one of the most intellectually challenging. It is even more critical when scaling from gram to metric ton scale. At that stage, it cannot be emphasized enough that optimal manufacturing routes must be time- and cost-effective, efficient, safe, and environmentally sustainable. Designing such routes requires scientific intuition, as well as depth and breadth of knowledge in synthetic chemistry. Since the 1960s, synthetic chemists have increasingly relied on computers to suggest the most promising synthetic routes and help plan their execution (Corey and Wipke, 1969; Cook et al., 2012). Computer-Assisted Synthesis Planning (CASP) primarily involves retrosynthesis, condition recommendation, and forward reaction prediction (Struble et al., 2019). Retrosynthesis aims at generating feasible pathways starting from the target compounds and ending with building blocks that can be easily acquired. Traditionally, it has been achieved using a knowledge-based approach, which iteratively applies *a priori* expert knowledge (including reaction templates and constraints) encoded as rules or heuristics (Marcou et al., 2015; Szymkuć et al., 2016). One example of retrosynthetic pathway prediction tools is Synthia™ (formerly Chematica) (Szymkuć et al., 2016; Grzybowski et al., 2018), which was used in a 2018 study to design multistep synthetic routes to eight structurally diverse targets with medicinal relevance that were successfully executed in the laboratory (Klucznik et al., 2018).

Despite its interpretability, the knowledge-based approach can be costly due to maintenance and expansion of knowledgebases and is not very applicable to novel chemistries (Kayala, 2011; Reng et al., 2018). Recent advances in deep/transfer learning have enabled the development of innovative approaches that can automatically learn from available data, suggest routes, and predict outcomes (Gao et al., 2018; Dai et al., 2020; Schwaller et al., 2021). Additionally, several hybrid approaches have been developed that implement rule-based algorithms to suggest possible reactions which are then ranked and selected using machine learning algorithms (Zhang and Aires-de-Sousa, 2005; Segler and Waller, 2017; Nicolau et al., 2020). The prediction of reaction conditions is helpful for the prioritization of safe and efficient reactions. The outcome of such predictions usually includes chemicals (e.g.: catalysts, reagents, and solvents) and physical properties (e.g.: pressure, temperature). Examples of such prediction models include expert systems (Marcou et al., 2015) and machine learning-based models (Gao et al., 2018; Walker et al., 2019; Maser et al., 2021). Forward reaction prediction helps validate each reaction step and identify by-products to facilitate purification. Additionally, the prediction of yield provides a measure of how efficient a reaction step or route is. Recently, several tools have been proposed that address the prediction of both reaction outcomes and yields (Coley et al., 2017; Haywood et al., 2021; Martinez et al., 2021). As with several other applications of predictive modeling, high-quality, comprehensively annotated data can be very scarce and sparse. Moreover, collected reaction datasets tend to omit less successful and failed reactions. However, these would provide more insights into the mechanisms and latent variables that can best describe the feasibility of chemical reactions and thus, improve prediction accuracy. The recently released Open Reaction Database (ORD) is an effort to promote the sharing of proprietary pre-competitive reaction data in a comprehensive yet structured format (Kearnes et al., 2021). The ORD allows users to upload, search, visualize, and eventually submit chemical reaction data through programmatic access and web interfaces. By adhering to those standards, researchers can contribute to the amount and diversity of high-quality data available to carry out diverse CASP projects.

Currently, several CASP tools are freely or commercially available. Most of them provide a graphical user interface that enhances user experience, with the capabilities of visualizing and interacting with the proposed reactions and pathways (See Table 2). So far, it is not immediately apparent whether rule-based or machine learning-based approaches consistently provide superior results. However, a significant advantage of machine learning-based tools is that they can be rapidly improved and scaled efficiently as more data become available. Additionally, they tend to be easier to generalize over a larger chemical space. This is especially the case for template-free reaction prediction models. The evaluation of predicted pathways is generally carried out manually by groups of chemists. In order to perform pathway evaluations in a systematic, reliable, and consistent manner, automated and scalable frameworks need to be designed, which could be improved with the availability of additional data. Mo et al. (2021) introduced a data-driven approach to evaluate the relative strategic levels of retrosynthesis routes. The resulting tree-LSTM model, built on 238K routes from patents, could not only recognize but also cluster similar pathways. More recently, PaRoutes was introduced as a framework for comparing the quality and diversity of predicted synthetic pathways (Samuel and Bjerrum, 2022). The authors suggested metrics that could serve as methods for comparing predictions. It is envisaged that the significant efforts in this space will help to define metrics and workflows for the comparative evaluation and prioritization of predicted pathways, which would eventually point to cases where one prediction algorithm or tool performs comparatively better than its peers. Moreover, it could enhance the identification of innovative synthetic routes.

**TABLE 2:** Examples of software tools and resources used in the Make phase of the DMTA cycle.

Name	Description	References/Examples
ASKCOS	Machine-learning based; single- and multistep retrosynthesis; condition recommendation; forward reaction outcome and evaluation	Struble et al. (2019)
Synthia™ (Chematica)	Manual and computer-aided retrosynthesis; User-defined rules and filters; direct link and metadata to commercially available and known building blocks	Grzybowski et al. (2018); Klucznik et al. (2018)
IBM-RXN	Molecular Transformer-based models for retrosynthesis, and forward reaction prediction	Schwaller et al. (2021)
ICSYNTH	Retrosynthesis analysis; machine-learned chemical rules; not limited to organic reactions	Bøgevig et al. (2015)
Spaya	Machine learning-based tool for full retrosynthetic analysis	Iktos, (2023b)
Reaxys	Predictive retrosynthesis with deep neural networks train on Reaxys data	Elsevier, (2023)
ChemFinder™ Ultra	Database management and structure search; retrieval of chemical and biological data (documents, structures, reactions, properties, etc.); property calculation	Aldrich, (2023)
CAS SciFinder^n^	Search engine; retrieval of chemical data (structures, reactions, properties, etc.); linked to the CAS Content Collection™	CAS, (2023)
ReactionSage™	AI-based reaction pathway prediction; retrosynthesis, and forward reaction prediction	KEBOTIX, (2023)

The synthesis of target compounds requires that starting materials and reagents are available. Electronic catalogs and databases containing structural information and metadata about building blocks and reagents can be linked to CASP platforms (e.g.: ASKCOS (Coley et al., 2019)). Such libraries can be maintained manually, or by service providers such as eMolecules (eMolecules, 2023), and Chemspace (Chemspace, 2023), the latter of which is the world’s largest available compound catalog containing over 1.6 billion in-stock and make-on-demand building blocks. Another strategy used by chemists is the enumeration of chemical virtual libraries, where complex molecules are virtually created, using cheminformatics tools, by applying selected, easily reproducible chemical reaction schemes on available building blocks. The resulting compounds can then be filtered based on several criteria (max price, delivery time, properties, predicted activity), and then synthesized. An example of such virtual libraries is the Proximal Lilly collection, which provides chemists with a diverse collection of compounds that can be synthesized in-house (Nicolaou et al., 2016). Once obtained, the compounds can be tested in various experimental assays. Currently, cheminformatics platforms are being developed to assist chemists from planning to compound ordering to automation of synthesis (Schwaller et al., 2021; IBM, 2023).

### 3.3 Test

**FIGURE 4:**
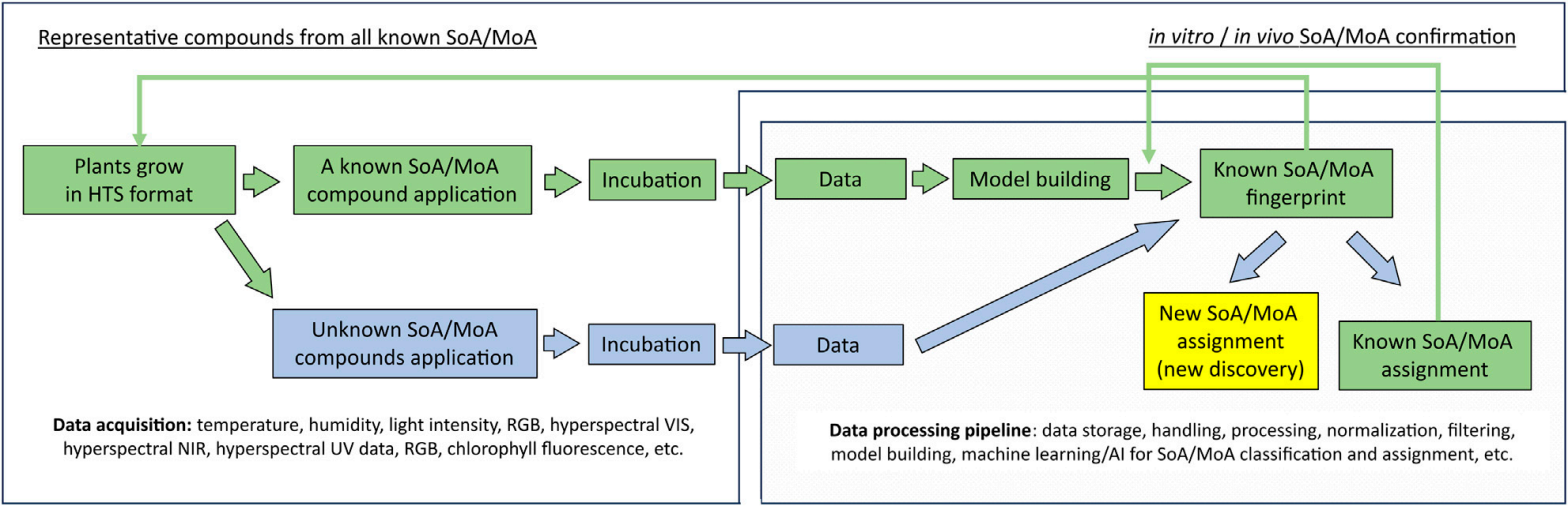
Workflow to identify herbicide SoA/MoA for screening compounds using high-throughput (HTS) assay, data rich imaging technologies, and Machine Learning/AI. Green colored workflow: compounds with known site of action (SoA)/mode of action (MoA) are used to build and validate the model to generate SoA/MoA fingerprints; Blue colored workflow is for new compounds with unknown SoA/MoA handling. Arrow indicates the workflow direction. After the model building and validation are completed with representative compounds from each known SoA/MoA, when new compounds with unknown SoA/MoA are tested and analyzed using this workflow, the compounds can either be assigned as new or unknown SoA/MoA, which warrants further efforts to use the DMTA cycle to discover novel herbicide with new mode of action; or be assigned as known SoA/MoA, after these assignment are confirmed by *in vitro* and/or *in vivo*, these data can be used for further model refinement.

In agrochemical discovery, substantial amounts of data from a plethora of assays run on target and non-target species are obtained for further analysis. It is thus particularly important to enhance testing capabilities as well as data collection. The synergistic interaction between cheminformatics and biological tests in agrochemical discovery has not been commonly discussed, yet it has been essential. Data from medium and high throughput assays, such as *in vitro* enzyme assays, cell-based assays, metabolomics/genomics assays, and *in vivo* whole organism plate-based assays have been used as input for cheminformatics tools. The improvement of computational power, data storage capacity, data analysis capability, and the integration of these three in low-cost cloud computing services (e.g.: Amazon Web Services™ cloud computing platform (Amazon Web Services, 2023)) for cheminformatics tools have enabled new generation of data collection with existing assays, especially in whole organism level assays. For example, data rich hyperspectral/multispectral imaging (Thomas et al., 2018; Paulus and Mahlein, 2020; Klie et al., 2022) and video-based chemobehavioral phenotyping (Henry and Wlodkowic, 2020) provides enriched data to further enhance cheminformatics development such as building more sophisticated models and enabling the training of AI predictive models (Ozdemir and Polat, 2020). On the other hand, new and powerful predictive models can further support the automation in non-destructive data collection, mode-of-action prediction, and so forth, which can further increase the test throughput potential and derive extra value from each test, especially for *in vivo* tests (Mishra et al., 2017; Klie et al., 2022). For example, Klie et al. recently disclosed a workflow that can be used to predict herbicidal site of action and/or mode of action of novel chemistries using classical machine learning and/or AI (See Figure 4).

### 3.4 Analyze

The Analyze phase of the DMTA cycle is a continual process during the entirety of a project’s timeline. The analyses are used in the Design phase to help determine what compounds to synthesize, in the Test phase to help evaluate and plan additional tests, and in an oversight role to determine whether to continue a project or not. Cheminformatics plays a significant role in helping the researcher answer key questions in all these phases. The following subsections describe four critical aspects of the analyze phase and illustrate their overall impact in the DMTA cycle, while providing a brief description of several tools that enhance the analyses (See Table 3). These include structure classifications, predictive modeling, SAR visualizations, and metabolomics.

**TABLE 3:** Examples of relevant resources, cheminformatics software, and machine/deep learning tools utilized in the analyze phase of the DMTA cycle in agrochemical discovery. Abbreviations: Support vector regression (SVR), Liquid Chromatography – Mass Spectrometry (LC-MS), Graph Neural Network (GNN), Retention Time (RT), Deep Graph Learning (DGL), Natural Products (NP).

Name	Description	References/Examples
*Structural classifications tools*		
LeadScope	SAR analysis and visualization tool, with a focus on toxicological data	Roberts et al. (2000)
DataWarrior	General purpose SAR tool	Sander et al. (2015)
Pipeline Pilot	Data pipeline tool; capabilities for various *ad hoc* analyses	Dassault Systèmes SE, (2023)
KNIME	Data pipeline tool; capabilities for various *ad hoc* analyses	Berthold et al. (2008)
OpenEye Toolkit	Molecular toolkit; Low-level API tools custom structure analyses	OpenEye Scientific (2023a)
RDKit	Molecular toolkit; Low-level API tools custom structure analyses	RDKit, (2023)
*Structure-Activity-Relationship Visualizations*		
DataWarrior	General purpose SAR tool	Sander et al. (2015)
StarDrop™	Includes multi-parameter optimization and SAR tools	Optibrium, (2023)
TIBCO Spotfire^®^	Lead Discovery collection adds extensive cheminformatics capabilities, including predictive analytics	TIBCO, (2023)
*Cheminformatics and AI-enabled Metabolomics*		
Peakonly	DL-based model for LC-MS peak detection and integration	Melnikov et al. (2020)
ChromAlignNet	DL-based tool for peak-alignment of GC-MS data	Li and Wang (2019)
CFM-ID	Hybrid (AI-, rule-based) tool for LC-MS spectra prediction, peak annotation, and metabolite identification	Wang et al. (2021a), Djoumbou-Feunang et al. (2019b)
3D-MolMS	Tandem MS Spectra prediction	Hong et al. (2023)
MassFormer	Tandem MS Spectra prediction	Young et al. (2023)
SIRIUS	Computational platform for tandem MS data-based analysis of metabolites; provides molecule search, and class prediction capabilities	Dührkop et al. (2019)
MESSAR	Automated tool for metabolite substructure recommendation from tandem mass spectra	Liu et al. (2020)
ClassyFire	Structural classification of small and large molecules	Djoumbou-Feunang (2016)
NP-Classifier	DNN-based structural classification of natural products	Kim et al. (2020)
BioTransformer	Hybrid, comprehensive tool for metabolite prediction and identification in humans, gut microbiota, and environmental microbiota	Djoumbou-Feunang et al. (2019a)
ADMET Predictor	Machine learning-based prediction of human metabolites	SimulationsPlus, (2023)
QSAR Toolbox	AI-based prediction of chemical products from abiotic transformations and metabolism (microbial, rat liver S9, skin)	QSAR Toolbox, (2023)
OASIS Times	AI-based prediction of chemical products from abiotic transformations as well as *in vitro* (gut, lung, rat liver S9) and *in vivo* (rat) metabolites	OASIS, (2021)
GLORYx	Machine learning-based prediction of human metabolites	de Bruyn Kops et al. (2021)
MetaTrans	Deep-learning-based, rule-free tool for prediction of small molecule metabolites in humans	Litsa et al. (2020)
Retip	ML-based retention time prediction	Bonini et al. (2020)
GNN-RT	GNN-based liquid chromatography retention time prediction	Yang Q. et al. (2021)
DeepCCS	Deep Learning tool for the prediction of collision cross-section values	Plante (2019)
Spectral Databases	Spectral databases commonly used for metabolite identification	(NIST, (2023); Guijas et al. (2018); Wang et al. (2021b); Mehta, (2020); Wishart et al. (2018); Wang et al. (2016)
*Programming libraries and cheminformatics tools for predictive modeling*		
Scikit-learn	General Python-based programming library	Pedregosa et al. (2011)
PyTorch	General Python-based programming library for deep learning, including explainable DL	PyTorch, (2023)
Tensorflow	General Python-based programming library for deep learning, including explainable DL	Abadi et al. (2015)
DeepChem	Python-based programming library for deep chemistry	Ramsundar et al. (2019)
Chemprop	Python programming package implementing Message Parsing Neural Networks (MPNN) for the prediction of molecular properties as well as chemical reactions; provides uncertainty quantification capabilities	Yang K. et al. (2019)
DGL-Lifesci	Python programming library for graph neural network-based learning for chemistry and biology	Li Y. et al. (2021)
MolPMoFit	Transfer learning approach (and model) for molecular property (QSAR/QSPR) prediction	Li and Fourches (2020)
Chemformer	A Python library for molecular optimization, property prediction, reaction and retrosynthetic prediction	Irwin, Dimitriadis et al. (2022)
DESlib	A Python library for dynamic classifier and ensemble selection	Cruz et al. (2020)
SHAP	A Python programing library for Shapley Additive exPlanations	Lundberg and Lee, (2017); Rodríguez-Pérez and Bajorath, (2021)
Alibi Explain	Implements several algorithms for inspecting and explaining machine learning models	Klaise et al. (2021)
GNN-Explainer	A Python library for the explanation of GNN-based predictions	Ying et al. (2019)
CIME	A library for web-based exploratory analysis of chemical model explanations	Humer et al. (2022)

#### 3.4.1 Structural classifications

Structure classification tools allow the partitioning of compounds into groups that can be used in a variety of visual and statistical tools to highlight areas of particular interest. This capability is at the heart of commercial software tools such as LeadScope (Roberts et al., 2000) and open-source software tools such as DataWarrior (Sander et al., 2015).

Some of the most common classification approaches include the identification of ring systems and frameworks, and clustering based on structural fingerprints. The compounds in each cluster can be further classified by determining the “Maximum Common Substructure”, i.e., the largest substructure that is found in each compound in the cluster. These classifications tend to be “unsupervised”, driven solely by the nature of the structures on hand, and thus are easy to accomplish with the use of modern cheminformatics toolkits (See Table 3).

A semi-manual approach, R-Group Decomposition (RGD), involves the identification of specific core structures in a molecule set, then determining the substituents that are attached in specific locations on the core (Agrafiotis et al., 2011; Naveja and Vogt, 2021). This technique usually involves an iterative analysis in order to describe as many compounds in the project as possible. In the end, the researcher is left with a set of molecular partitions and descriptors that generally align with the synthetic sources of the molecules. One important use of the resulting RGD table is to track the specific compounds that have been made and which of these have been tested in which assays. It also helps to quickly spot and track gaps in the already designed libraries, which is particularly important given that most researchers work on many projects simultaneously and over many years.

#### 3.4.2 Predictive modeling

**FIGURE 5:**
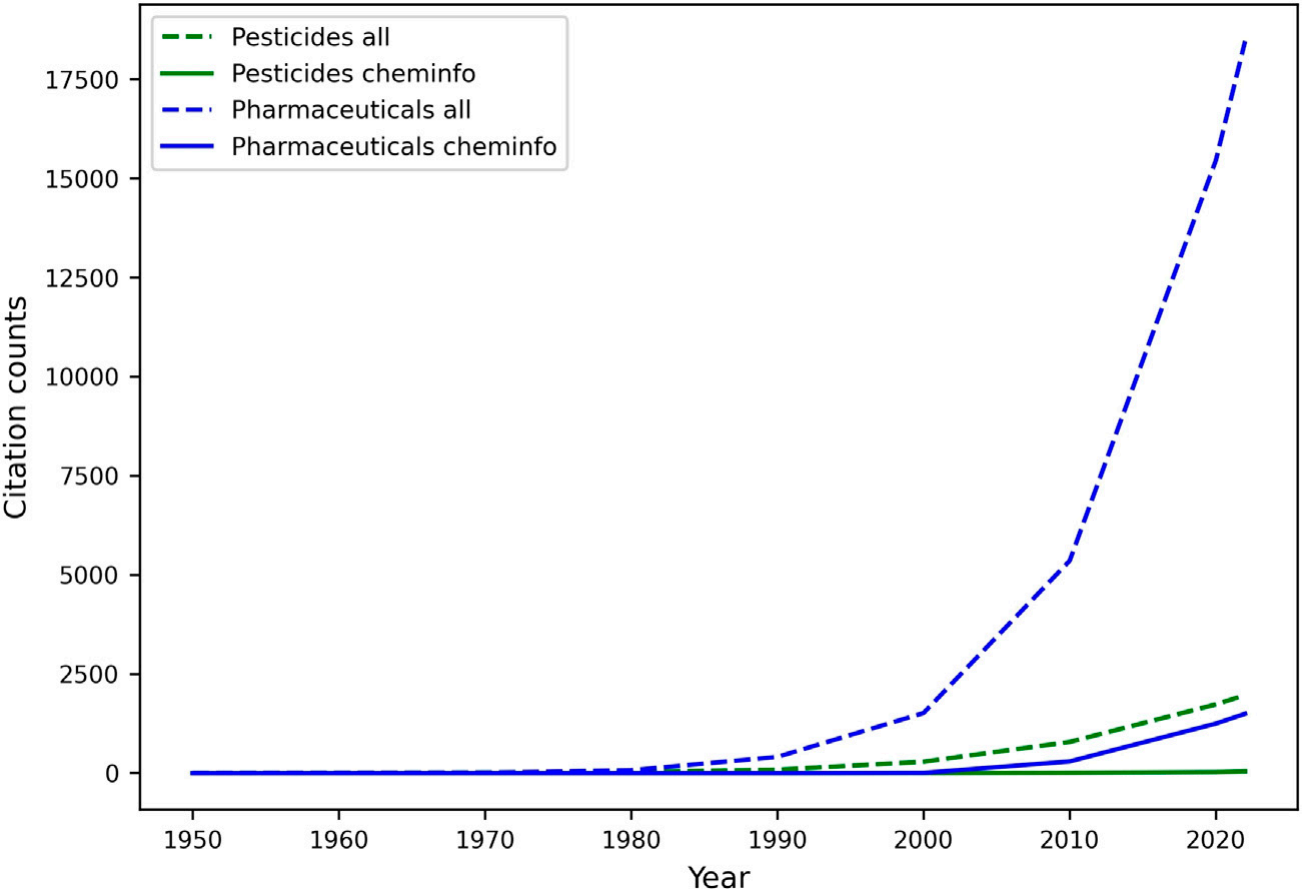
Comparative analysis of machine learning and cheminformatics-related publication counts for pesticide and pharmaceutical discovery. Publications (articles, reviews, reports, and dissertations only) were retrieved in May 2022 from the web of science literature database, upon mining the title and abstracts for specific keywords. The search for cheminformatics papers was limited to publications containing either of the keywords in the title and/or abstract: “cheminformatics” (or a derivative), ”QSAR”, “QSPR”, “QSBR”. The list was expanded to include papers from selected scientific journals whose title contains either “cheminformatics” (e.g.: journal of cheminformatics) or “QSAR”. ML papers included various (groups of) keywords related to ML tasks, and metrics. The keyword “all” refers to publications including machine learning- and cheminformatics-related terms. Keywords describing the molecule class included drug, pharmaceutical, agrochemical, pesticide, insecticide, herbicide, fungicide, nematicide, and their derivatives.

One of the most important activities in the DMTA cycle, and in the analyze phase, involves the study of quantitative relationships between molecular structures and various endpoints, including but not limited to biological activity (QSAR), physicochemical properties (Quantitative Structure Property Relationships; QSPR), and biodegradation (Quantitative Structure Biodegradation Relationship; QSBR). Leveraging diverse datasets generated throughout the test phase, among other sources, machine learning, and especially predictive modeling, have gradually matured over the last few decades into an essential component of discovery and regulatory processes for pharmaceuticals and agrochemicals (Naik et al., 2009). They both deliver mathematically sound, reliable, cost- and time-efficient, and more accessible “*in silico* assays” that can predict relevant endpoints, and be automatically improved with increasing data, in an adaptive environment (J. C. Dearden, 2016; Yang K. et al., 2019; Shen and Nicolaou, 2019). Most innovation in this space has occurred in pharmaceutical research, and agrochemical research has followed suit. Unfortunately, as illustrated by a relatively low number of related publications (See Figure 5), the adoption of an AI-driven discovery paradigm is not nearly as rapid in the relatively smaller space of agrochemical discovery, leaving untapped an increasing reservoir of innovative opportunities to accelerate research and development. Yet, urgent needs for novel and safer crop protection agents, along with the resolutions of regulatory agencies (e.g.: U.S. EPA (United States Environmental Protection Agency, 2023), EFSA (European Food and Safety Agency, 2023)) to aggressively reduce animal testing (Barlow et al., 2009), highlight the need for predictive tools that provide different lines of evidence and support the use of New Approach Methodologies (NAMs) in various scientific tasks, such as chemical risk assessment (U.S. EPA, 2021; Kavlock et al., 2018).

Ideally, a crop protection agent must display optimal properties with respect to efficacy, metabolic stability, activity spectrum, uniqueness of its mode of action, and sustainability, among other parameters. High-Throughput Screening (HTS) is a key component of the discovery pipeline that provides scientists with diverse types of data exploitable for decision-making. In contrast to drug discovery, however, most screening assays in “agchem” discovery are phenotypic and run against whole organisms, especially in preliminary stages, when the target site is unknown. An advantage of such assays is that they incorporate the cellular complexity of biology as they highlight molecules that are both intrinsically active and bioavailable (Bender and Cortés-Ciriano, 2021a). However, they only provide little insight on the mode of action, which may be species-dependent (FRAC, 2023; HRAC, 2023; IRAC, 2023), and very little on the fundamental mechanisms that make a compound more or less active or completely inactive (e.g.: ADME properties), thus posing challenges for subsequent optimization. Bottlenecks resulting from these limitations include, among others, poor translation of activity against selected targets from the greenhouse to the field, limited systemic activity, and discrepancies between *in vivo* and *in vitro* activities (Zhang et al., 2018) Since activity, ADME-Tox, environmental fate, and other relevant mechanisms are influenced by the molecule’s physicochemical properties (e.g.: lipophilicity (LogD), water solubility (WS), UV stability, pKa), QSPR-based tools that rapidly and accurately predict such properties are indispensable for rapid exploration of the immense chemical space, efficient selection of promising candidates, and decision making. For instance, physicochemical property prediction tools support the estimation of ag-likeness, typically defined with various degrees of specificity. Herbicides and sap-feeding insecticides, which need to be transported through the plant’s xylem typically display high WS and low LogD, while chewing insecticides display high LogD and low WS that limit their uptake by, and mobility within plant leaves (Zhang et al., 2018). Commercial fungicides, however, occupy a relatively broader range with respect to those properties. Several guidelines have been proposed by Tice (2001), Zhang et al. (2018), and others (Hao et al., 2011; Avram et al., 2014) to assess ag-likeness based on various molecular (e.g.: constitutional, physicochemical) descriptors. Examples of most commonly used open-source cheminformatics packages for the computation of such molecular descriptors include, among others, RDKit (RDKit, 2023), Mordred (Moriwaki et al., 2018), the PaDEL-Descriptor software (Yap, 2011), and the Chemistry Development Kit (CDK) (Willighagen et al., 2017). While these tools typically provide a diverse set of descriptors, they often either lack certain physicochemical properties used in ag-likeness rules (e.g.: UV-stability, pKa) or provide different implementations compared to those used in the rules (e.g.: XLogP vs. ALogP vs. MLogP). Freely available and interactive web platforms such as InsectiPAD (Chen-Yang et al., 2019), FungiPAD (M.-y. Wang et al., 2019), and HerbiPAD (Huang, 2020) provide capabilities to explore pre-computed physicochemical properties and evaluate pesticide-likeness of chemicals. However, these are limited to only a few hundred chemicals and cannot be easily integrated into *in silico* workflows. OPERA (Mansouri et al., 2018) is an open-source/open data, standalone, limited collection of QSPR/QSAR models that predict several toxicity (e.g.: androgen receptor activity) and environmental fate (biodegradation half-life) endpoints, along with other fate-related properties (e.g.: water solubility). In general, the combination of QSPR-, ag-likeness-, and other endpoint prediction models (e.g.: QSAR), can guide stepwise virtual screening programs, as demonstrated in several studies (Oršolić et al., 2021; Lewer et al., 2022). These tools provide much needed capabilities for ligand-based discovery, especially in early stages, where targets and/or modes of action are unknown.

**FIGURE 6:**
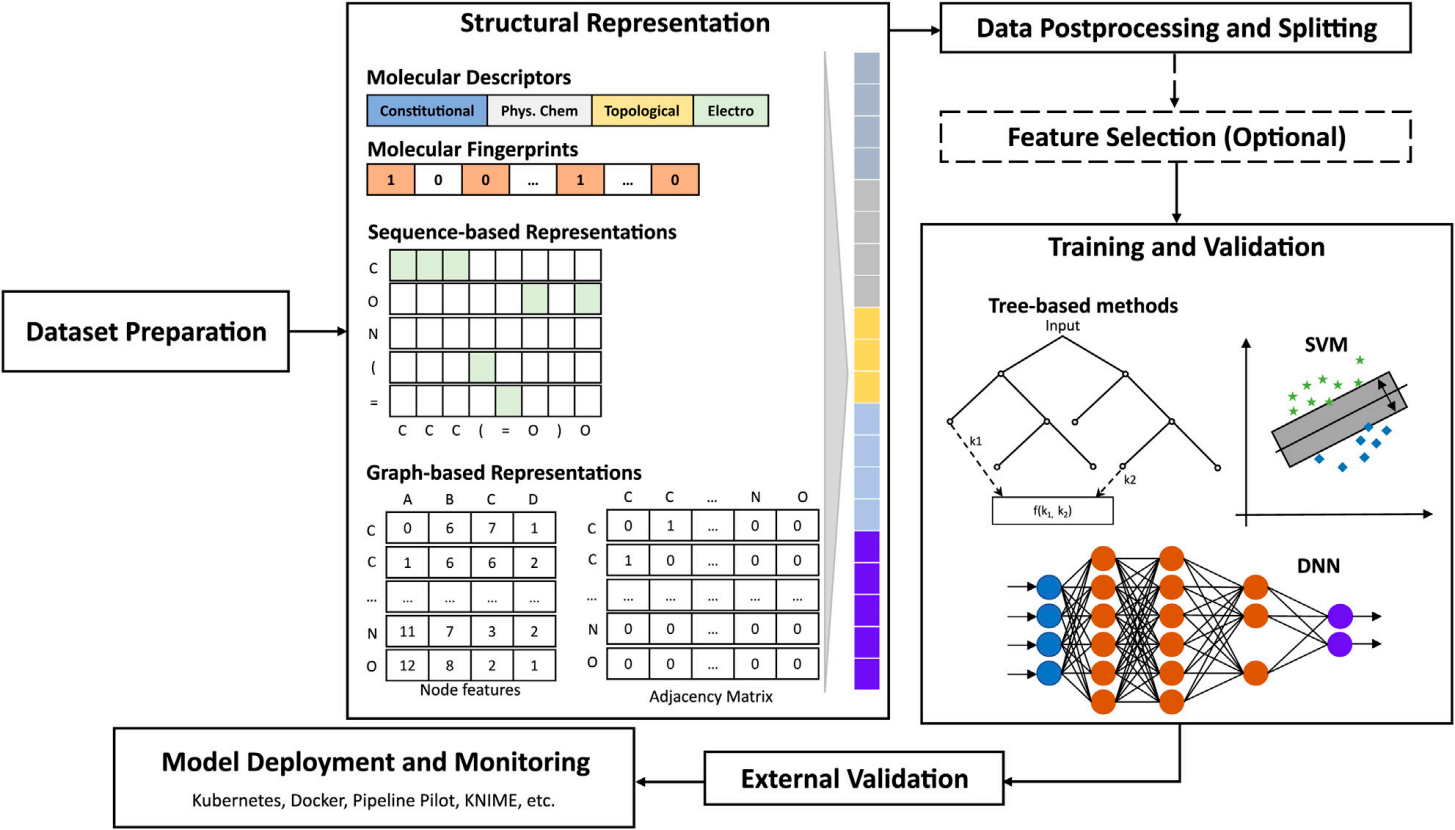
Schematic overview of the QSAR/QSPR modeling and deployment process.

At later stages of the discovery pipeline, leads must still be optimized with respect to activity against target and non-target species (Martin et al., 2017), favorable/unfavorable modes of action (Kienzler et al., 2017), efficacy, metabolism in target and non-target species (Clark, 2018; Diéguez-Santana et al., 2022), abiotic degradation, and (eco-)toxicity (Devillers et al., 2015; Venko et al., 2018), among other parameters. Some examples include the implementation of a 3D-QSAR approach for the prediction of acetylcholinesterase inhibition of pesticides (Lee and Barron, 2016), the integration of mode of action information into classification and regression QSAR models for the prediction of acute toxicity in honeybees (Carnesecchi et al., 2020a), and the development of OECD-compliant models that accurately predict biodegradation rates of organic compounds (Tang et al., 2020). Additionally, QSAR/QSPR models in later stages could enhance for instance, the improvement of activity and ADME-Tox profiles, and the promotion of more sustainable crop protection agents with minimal risk of resistance (Oršolić et al., 2021). Given the structural differences between pesticides and drugs, it is worth noting that the QSAR/QSPR tools used at each stage of the pipeline, should be either generalizable enough, or at the very least, applicable to the local or global agchem space of interest. Unfortunately, most predictive models available either commercially or open-source are trained on datasets significantly biased towards drugs and drug-like molecules. Moreover, many of the relevant published studies focus on small samples (<500 compounds), thus describing local models. Consequently, crop protection discovery scientists are often forced to a tradeoff between using such tools with less certainty, adapting them towards agrochemicals, or building entirely new predictive models (See Figure 6).

Developing valid, OECD-compliant (Benfenati et al., 2011; Belfield et al., 2023; OECD, 2023) predictive models depends on several key factors: 1) high-quality datasets; 2) proper mathematical representations of molecules that capture key elements essential for the learning task, and powerful computational methods to capture the complex patterns of association between the molecular representations and target endpoints; 3) rigorous performance evaluation criteria, and 4) adequate methods for explainability and uncertainty estimation. In the following, “QSAR” is used as a general term for the quantitative relationship between chemical structures and relevant endpoints (activity, properties, biodegradability, toxicity, etc.).

##### 3.4.2.1 High-quality datasets

Predictive models typically require training on sufficiently large and diverse datasets. Modern high-throughput techniques for the measurement of proxy points (e.g.: LogD), along with increasingly powerful automated text mining and data extraction technologies (Han et al., 2010; Tarasova et al., 2019; NextMove Software, 2022; Shavalieva et al., 2022) have enhanced the acquisition of physicochemical and biological data through internal laboratories (Zhang et al., 2018), CROs, and large-scale data mining projects, sometimes resulting in the publication of FAIR-compliant data (Wilkinson et al., 2016). However, many data-related issues still impede the development of accurate “Ag-adapted” models. These issues include: 1) relatively smaller number of data collected through whole organism assays; 2) relatively smaller coverage of ag-like compounds and Ag-relevant assay data (e.g.: non-target toxicity, bioremediation, plant metabolism, etc.) in public and private databases (Lewis et al., 2016; Gaulton et al., 2017; Williams et al., 2017; Wishart et al., 2017; Kim H. et al., 2021); and 3) the inconsistencies in experimental settings, which are often not taken into consideration during data curation. These limitations can impede the modeling of complex biochemical characteristics or activities and limit the exploration of algorithms such as deep neural networks that require vast amounts of high-quality data. When applicable, scientists often implement different techniques to circumvent these obstacles, that include but are not limited to oversampling/undersampling (Idakwo et al., 2020), cross-validation and cross-testing (Korjus et al., 2016), ensemble learning (Hung and Chang, 2021), data augmentation (Cortes-Ciriano and Bender, 2015; Bjerrum, 2017), transfer learning (Shen and Nicolaou, 2019), multi-task learning (Xu et al., 2017; Martin and Zhu, 2021), representation learning (Kim S. et al., 2021), and self-supervised learning (Dillard, 2021).

##### 3.4.2.2 Mathematical representations and machine learning methods

The hypothesis underlying QSAR studies is that structurally similar molecules tend to behave similarly and to exhibit similar physicochemical properties. Therefore, the selection of molecular representations that are predictive of the molecular property endpoint (e.g.: activity, physicochemical property) is critical for any machine learning task (Bender and Cortés-Ciriano, 2021b). Ideally, such representations shall efficiently express the structural composition of, and subtle nuances between molecules, in a faithful and consistent manner (Chuang et al., 2020). Moreover, interpretable representations would facilitate the human understanding of relevant patterns learned. One can distinguish between fixed, more interpretable representations (e.g.: whole molecule descriptors, atomic descriptors, quantum properties, dictionary- and hash-based fingerprints), and learned, more parsimonious, less interpretable representations (e.g.: convolution- or sequence-based embeddings). Readers are referred to reviews that provide detailed descriptions, comparisons, and applications of structural representations for QSAR (Shen and Nicolaou, 2019; David et al., 2020).

Traditional machine learning approaches typically involve a challenging combinatorial optimization process which consists of selecting a set of most relevant features or feature combinations from a variety of pre-calculated, fixed representations (Goodarzi et al., 2012; Mao et al., 2021) that serve as input to build predictive models that implement one or many algorithms (e.g.: Random Forest, SVMs) (Wu et al., 2020; Yang L. et al., 2021). Molecular fingerprints are often used in addition or as alternatives to the common 2D/3D (e.g.: constitutional and topological) descriptors. In fact, several studies have demonstrated that models based exclusively on fingerprints can outperform 2D/3D-descriptor-based models on various tasks (Venkatraman, 2021). For instance, Li et al. (2017) developed binary and tertiary classification models to predict pesticide aquatic toxicity against rainbow trout and *Lepomis* species, using only fingerprints. The best models implemented SVMs or ANNs on MACCS (Durant et al., 2002) or Graph-only fingerprints and achieved accuracies of 0.89 or higher. Examples of open-source packages that compute molecular fingerprints (FPs) include RDKit, CDK, and the PaDEL descriptor software. Limitations of molecular fingerprints include, among others, limited applicability domain of dictionary-based FPs, and sparsity, possible data loss, and bit collision for hash-based FPs. Moreover, the best fingerprint type can vary depending on the problem, and even between different train-test splits of the same dataset (Sandfort et al., 2021). To address these, several methods have been proposed, such as variants of circular fingerprints, and the combination of various fingerprint features (Capecchi et al., 2020; Sandfort et al., 2021). Fingerprints and molecular descriptors are by no means mutually exclusive. In fact, in many cases, the combination of both types of descriptors can lead to better results (Shi et al., 2018; Tian et al., 2021).

The success of ANNs in computer vision and natural language processing (NLP) in the 2000s has renewed interest in these algorithms, which had fallen out of favor due to many practical issues (e.g.: speed, overfitting, memory requirements). As early as 2008, Sparks *et al.* proposed a new ANN-based QSAR approach capable of suggesting structural modifications that dramatically improved the biological efficacy of Spinosyn analogs (Sparks et al., 2008), where other machine learning methods had failed. This innovation contributed to the design and registration of Spinetoram, a semi-synthetic insecticide. In 2015, Ma et al. (2015) demonstrated that deep neural networks trained using a set of atom pair-, and donor-acceptor pair-descriptors for molecular representation could routinely outperform the most-commonly used random forest models, with a 10% mean *R* ^2^ improvement (Ma et al., 2015) on various datasets. These success stories contributed significantly to the renewed interest in deep learning (DL) for chemistry (Chen et al., 2018). Chemical structures can be represented as graphs, or word sequences (e.g.: SMILES (Weininger, 1988)). Therefore, several algorithms have been developed to adapt DL algorithms, once prominent mostly in computer vision, NLP, and network modeling to the world of chemistry.

Prominent DL architectures for molecular property prediction include Graph Convolutional and Sequence-based models (See Figure 6). Graph convolutional networks (GCNs) take as input molecules encoded as graphs where nodes represent heavy atoms and edges represent covalent bonds between them (Gilmer et al., 2017; Lee and Min, 2022). Sequence-based models borrow ideas from NLP to utilize molecular representations such as SMILES strings for learning relationships between different parts of a molecule (akin to learning relationships between different words in a sentence) through recurrent neural network-based architectures such as LSTM and GRU (Goh et al., 2017). Several graph- and sequence-based DL algorithms have been implemented in DL packages such as DeepChem (Ramsundar et al., 2019), Chemprop (Heid et al., 2023), and DGL-LifeSci (Li M. et al., 2021). Over the last 5 years, significantly increased performances in molecular property prediction using DL relative to traditional machine learning models have been reported, with applications ranging from ADME-Tox modeling to bioactivity prediction (Montanari et al., 2019; Zhou et al., 2019; Feinberg et al., 2020; Stokes et al., 2020). More recently, several variations of graph-based and sequence-based (SMILES) algorithms have been demonstrated to achieve 14%–133% better performance than traditional machine learning algorithms in the prediction of relevant properties, in single- or multi-task settings (Honda et al., 2019; Yang X. et al., 2019; Sun et al., 2020).

A key advantage of DL algorithms is their capability of learning molecular representations in a supervised or unsupervised mode, with varying degrees of generalizability, depending on the intended use (Chuang et al., 2020). These representations, also known as molecular embeddings, can be trained using a variety of algorithms (e.g.: neural-based autoencoders, graph-neural networks, self-attention) to extract diverse information about physicochemical properties, structural properties, bioactivity, and other endpoints (Koutroumpa, 2023). The resulting DL models not only learn their own expert feature representations directly from the data, but they also learn how to weigh these features to deliver accurate predictions. Several frameworks have been implemented and published, which consist of pretraining models for sequence- or graph-based molecular representations in a self-supervised or unsupervised framework, using large unlabeled datasets (e.g.: ChEMBL (Gaulton et al., 2017), ZINC (Irwin and Stoichet, 2005)). The models can then be fine-tuned for more specific tasks. This methodology is particularly amenable to transfer learning, which has been very well exploited in both graph computing and NLP spaces. For instance, Ashtawy *et al. (* Ashtawy et al., 2021 *)* pre-trained a GNN molecular representation model that performs comparably or better than supervised models when fine-tuned over several ADMET related tasks. Li and Fourches proposed MolPMoFiT, a transfer learning approach based on self-supervised pre-training and task-specific fine-tuning for QSPR/QSAR modeling (Li and Fourches, 2020). MolPMoFiT was used to build predictive models for small datasets that showed comparable or better performances on several datasets compared to state-of-the-art D-MPNN, Random Forest, and other Feed Forward Network models. Lately, inspired by their success in NLP, attention-based transformer models (Honda et al., 2019; Irwin et al., 2022) have emerged as more powerful architectures for encoding molecular representations to predict reactions or properties. For example, to learn molecular embeddings, Irwin et al. (Irwin et al., 2022) pre-trained several Bidirectional Auto-Regressive Transformer (BART) models on >100 million datasets from the ZINC-15 dataset (Irwin and Stoichet, 2005). In a multi-task learning framework, the models were rapidly trained on several sequence-to-sequence (e.g.: direct synthesis) and discriminative (e.g.: activity) prediction tasks, yielding task-specific models with comparable or better performance compared to the baseline. Several other sequence-based (e.g.: Bidirectional Encoder Representations from Transformers (BERT), Siamese RNNs, and graph-based (e.g.: D-MPNNs) frameworks for representation and transfer learning have been developed and implemented to build predictive models with improved performances (Yang K. et al., 2019; Payne et al., 2020; Fernández-Llaneza et al., 2021) (See Table 4). Moreover, to leverage the advantages and alleviate the limitations of various molecular representations, it is common to build hybrid architectures by combining them, as illustrated by several recent publications (Hasebe, 2021; Li M. et al., 2021).

**TABLE 4:** Examples of key AI-driven algorithms and methods for prediction of molecular properties.

Class	Method description	References/Examples
Dynamic Selection	Techniques for dynamic selection of classifiers based on individual sample	Cruz et al. (2018)
Ensemble learning	Combination of multiple learners for performance improvement	Svetnik et al. (2003); Sheridan et al. (2016); Kwon et al. (2019); Davronov and Adilova, (2021)
Fully Connected DL	Fully connected deep learning network for Single-task QSAR analysis	Ma et al. (2015)
GCN	Multitask graph convolutional networks	Montanari et al. (2019)
Fully Connected DL	Fully connected deep learning network for Multi-task QSAR analysis	Kearnes et al. (2016)
GCN	PotentialNet family of graph convolutions for protein-ligand binding affinity	Feinberg et al. (2018); Feinberg et al. (2020)
GNN	Molecular Contrastive Learning	Wang et al. (2021c)
MPNN	Message passing neural networks for molecular property prediction	(Yang X. et al. (2019); Stokes et al. (2020); Chen et al. (2021); Heid et al. (2023)
Graph Transformer	Molecular encoding using hybrid MPNN-Transformer architectures	Rong et al. (2021)
NLP inspired	Autoencoder-based Molecular encoding and QSAR	Winter et al. (2019)
NLP inspired	Transformer based encoder model	Honda et al. (2019); Payne et al. (2020); Irwin, Dimitriadis et al. (2022)
NLP inspired	Transfer learning for NLP based classification tasks	Li and Fourches (2020)
NLP inspired	Siamese RNNs for QSAR Prediction	Fernández-Llaneza et al. (2021)
Active Learning	Retrosynthetic and combinatorial synthesis coupled with Active Learning	Konze et al. (2019)
Explainable Artificial Intelligence	Methods to provide interpretability to ML/DL models. These include approaches for explaining their predictions, quantifying their uncertainty, and estimating their applicability domains	Interpretability Ribeiro et al. (2016); Lundberg and Lee, (2017); Nori et al. (2019); Rodríguez-Pérez and Bajorath, (2021)
		Uncertainty estimation Liu et al. (2018a); Cortés-Ciriano and Bender, (2019); Gawlikowski et al. (2021); Zhong et al. (2022)
		Applicability domain Liu and Wallqvist, (2019), R. P. Sheridan, (2015); Schroeter et al. (2007); Supratik et al. (2018)

Training DNNs typically requires larger amounts of training data compared to traditional ML models. NLP-based algorithms can benefit from numerous augmentation methods, including SMILES randomization (Bjerrum, 2017; Arús-Pous et al., 2019) and other SMILES-derived encodings (Lambard and Gracheva, 2020) that can lead to improvements even in low-data regimes. Representation and transfer learning provide opportunities to lower data size requirements for the development of accurate predictive models. Increasingly popular techniques include one-shot-, few-shot-, and meta-learning, which learn rich molecular representations from relatively small datasets (Altae-Tran et al., 2017; Nguyen et al., 2020; Wang F. et al., 2021; Fernández-Llaneza et al., 2021; Guo et al., 2021) and self-supervised learning methods that leverage large unlabeled datasets (Dillard, 2021; Li P. et al., 2021). Finally, neural prediction models implement active learning approaches that can effectively sample the set of possible training candidates given a fixed training budget, thereby offering a systemic approach for exploring the data that is at the core of drug discovery research (Konze et al., 2019; Reker, 2019).

Overall, the methods mentioned above help modeling several endpoints of utmost importance in agrochemical discovery that have traditionally been difficult to tackle. For instance, the prediction of activity translation, which is typically limited to small datasets given the low number of molecules tested, especially in higher tiers, could be addressed using approaches that perform well in low-data regimes. The key methods discussed above are summarized in Table 4. For a comprehensive review of molecular representations and machine/deep learning methods used for molecular property prediction, readers are referred to other publications (Lo et al., 2018; Lo et al., 2019; Sun et al., 2020; Wieder et al., 2020; Mao et al., 2021; Dhamercherla et al., 2022).

##### 3.4.2.3 Rigorous performance evaluation criteria

The success stories referenced throughout this review highlight not only the importance of AI in crop protection discovery, but also the fact that so far, no single (ML or DL) algorithm or molecular representation (Sabando et al., 2021; Orosz et al., 2022) is found to be best suited for most modeling tasks. It is thus important to define means for adequate comparative evaluations of a model as it would provide a fair model assessment and facilitate the selection of the most suitable algorithms and approaches for future modeling tasks (Liu et al., 2018b). Examples of high-quality datasets that are used for training and comparative evaluations include, among others, the Tox21 (Huang et al., 2016; Mayr et al., 2016), PubChem BioAssay (Wang et al., 2012), and MoleculeNet (Wu et al., 2018) datasets, which are available either in raw formats or as encoded objects in various DL packages such as DeepChem (Ramsundar et al., 2019), Chemprop (Yang X. et al., 2019), and DGL-LifeSci (Li P. et al., 2021). It is highly desirable that such packages also include datasets for Ag-relevant molecular endpoints. In recent years, several comparative evaluations (with respect to accuracy, computational efficiency, etc.) of traditional and deep learning algorithms have been published (Jiang et al., 2021; Rao et al., 2021). In several experiments, traditional machine learning using traditional molecular representations approaches significantly outperformed deep learning models using unsupervised molecular representations, showing a different trend than studies referenced in the previous section. Interestingly, in a recent study combining less expensive traditional algorithms, such as Gaussian processes and random forests, Green et al. (Green et al., 2023) demonstrated that fixed [e.g.: ECFP (Rogers and Hahn, 2010]) or learned representations [e.g.: Mol2vec (Jaeger et al., 2018)] could often yield better overall results compared to fully deep-learning-based approaches, both for property- and ADMET-related predictive modeling tasks. The overall takeaway is that the potential of DL has not yet been fully exploited in chemistry. In contrast to other areas like computer vision, there is still a lot to uncover and prove. Moreover, traditional ML algorithms and molecular representation techniques will not be obsolete soon. It can be expected, as pointed by Bender and Cortés-Ciriano (Bender and Cortes-Ciriano, 2021a), that learned representations could become more useful in high-data regimes, whereas expert-chosen representations will probably remain more useful when data is scarce. Benchmarking would help establish guidelines in the setup and hyperparameter tuning, and in identifying trends that guide the selection of appropriate algorithms, molecular embeddings, and predictive models. Additionally, meta learning (Olier et al., 2018) can help understanding the relationships between the performance of ML algorithms and measurable properties, as well as selecting the best predictive models (Cruz et al., 2018; Olier et al., 2018; Cruz et al., 2020). Furthermore, given that ligand-based models are prone to false positives, more research is needed to develop algorithms that systematically identify gaps where the model learned a trivial relationship that is not generalizable.

##### 3.4.2.4 Explainability and uncertainty estimation of predictive models

**FIGURE 7:**
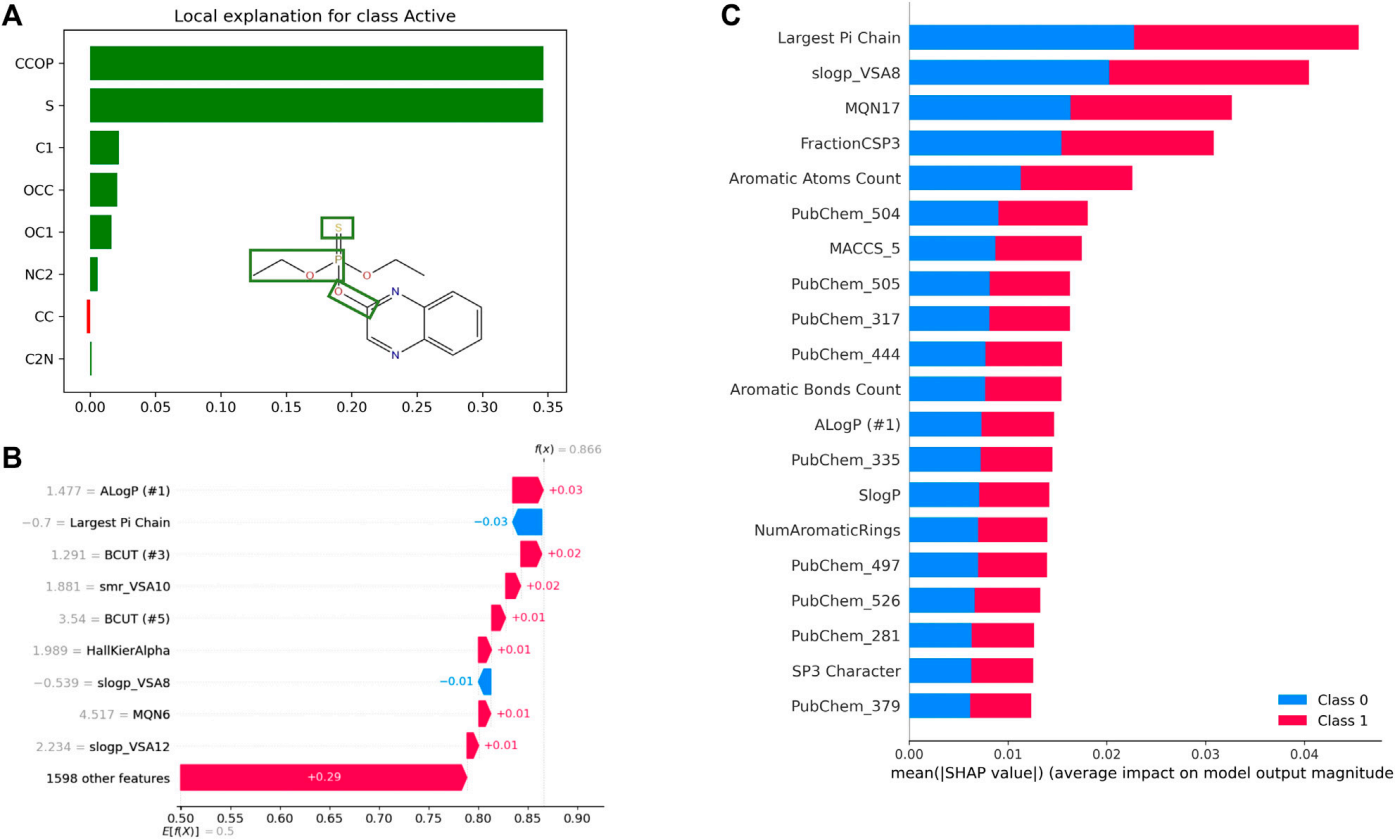
Examples of QSAR model explanations using feature attribution methods LIME and SHAP. **(A)** Explanation of Quinalphos’ predicted aryl hydrocarbon receptor (AhR) agonistic activity using LIME. The explanation displays the contribution of various tokens to the predicted output from an LSTM model trained on the Tox21 AhR dataset using SMILES representations. **(B)** SHAP Waterfall Plot to explain the contributions of various features towards AhR activity of a single molecule as predicted by a random forest model trained on the same dataset. This time, the model uses physico-chemical properties computed by RDKit as input features. For this example, the ALogP contributes the most towards predicted activity (class 1), while the Largest Pi Chain features contributes the most towards predicted inactivity (class 0). **(C)** Global interpretation of the AhR random forest model with SHAP. On average the “Largest Pi Chain” features contributes the most towards predicted activity and predicted inactivity.

Besides high performance (as measured by various metrics) and scalability, it is highly desirable that predictive models be explainable. The ability to assess the contribution of a molecule’s various structural features and physicochemical properties, among other features, towards quantitative or qualitative output variables is critical for designing, assessing, and optimizing molecules. Unfortunately, the black box nature of most ML (especially DL) approaches, makes it difficult to interpret the prediction from QSAR models, and thus, impedes their widespread adoption. In recent years, explainable AI (XAI) has been the focus of numerous drug discovery research projects (Jiménez-Luna et al., 2020b). In the area of QSAR, one can distinguish among feature-, atom/fragment-, compound-, and graph-based approaches for model explanation (Rodríguez-Pérez and Bajorath, 2021) (See Table 4). While atom-/fragment-based and graph-based approaches could, for instance, highlight substructures that contribute to soil degradation of a specific molecule, feature-based approaches could explain how specific molecular properties influence the toxicity against honeybees, for example. Commonly used methods include feature attribution and graph-convolution-based methods. Feature attribution methods, such as SHAP, LIME, and DeepLIFT, determine the importance of every input feature towards a prediction (See Figure 7). Various subgraph identification, and attention-based approaches have been developed to provide explainability to DNN models (Karpov et al., 2020; Weber et al., 2021). For instance, GNN-explainer, which provides explanations for every graph-based machine learning, was able to correctly identify several functional groups known to be mutagenic to *Salmonella typhimurium* (Ying et al. 2019). In recent years, several benchmarks have been published for comparing the interpretability of various XAI methods using traditional (e.g.: Random Forest, SVMs) and deep learning models (Sanchez-Lengeling et al., 2020; Klaise et al., 2021; Matveieva and Polishchuk, 2021).

The interpretability of a model can not only provide insights into the relationship between features and the modeled outcome, but also helps to select the best features to model similar tasks, resulting in better performance. However, as recommended by Muratov et al. (2020), model explanations must be used with caution. Scientists should only be confident in a predictive model if it is generalizable enough to perform well on unseen data, and the molecules of interest are within the model’s domain of applicability. It is, therefore, important that the predictive model be deployed along with tools or capabilities to define its domain of applicability for the assessment of compounds of interest, and to estimate the uncertainty of its predictions. Several approaches (e.g.: ensemble, probabilistic, and distance) that are applicable to different types of machine learning algorithms have been developed to quantify prediction errors and estimate applicability domains (Schroeter et al., 2007; Cortés-Ciriano and Bender, 2019; Gawlikowski et al., 2021). As demonstrated by Zhong et al. (2022), uncertainty estimation can also be used to increase the applicability domain of QSAR models, which is critical, especially in low-data regimes. Overall, it is believed that implementing methods for uncertainty estimation and model explainability could help tackle some of the most challenging, unaddressed problems, such as the prediction of activity translation and the prioritization of molecules between different experimental tiers, as the number of datapoints becomes increasingly smaller and more realistic experimental settings are employed for testing, thus increasing the complexity of modeling tasks.

#### 3.4.3 Structure-activity-relationship (SAR) visualizations

Adding information from our structure classifications (e.g.: R-group decomposition described above) and predictive models to any plot of biological activity automatically gives rise to SAR plots. It is now possible to look for themes and features that drive towards the desired activity profiles. Modern data visualization tools such as Tibco Spotfire with Lead Discovery (Perkin Elmer) (Elmer, 2023) and Tableau (Tableau Software, 2023) make it easy to construct interactive displays that allow the researcher to explore the connections between the structural features and classes, and the biological data. There are several good examples of additional SAR visualizations in the literature including SARNEA (Lounkine et al., 2010), SAR Matrices, which can overcome the inflexibility of R-group decomposition (Yoshimori et al., 2019), and SAR Maps (Agrifiotis et al., 2007). SAR Matrices can support bioactivity prediction, and large-scale database building for analog searching, among other applications (Yoshimori and Bajorath, 2020).

**FIGURE 8:**
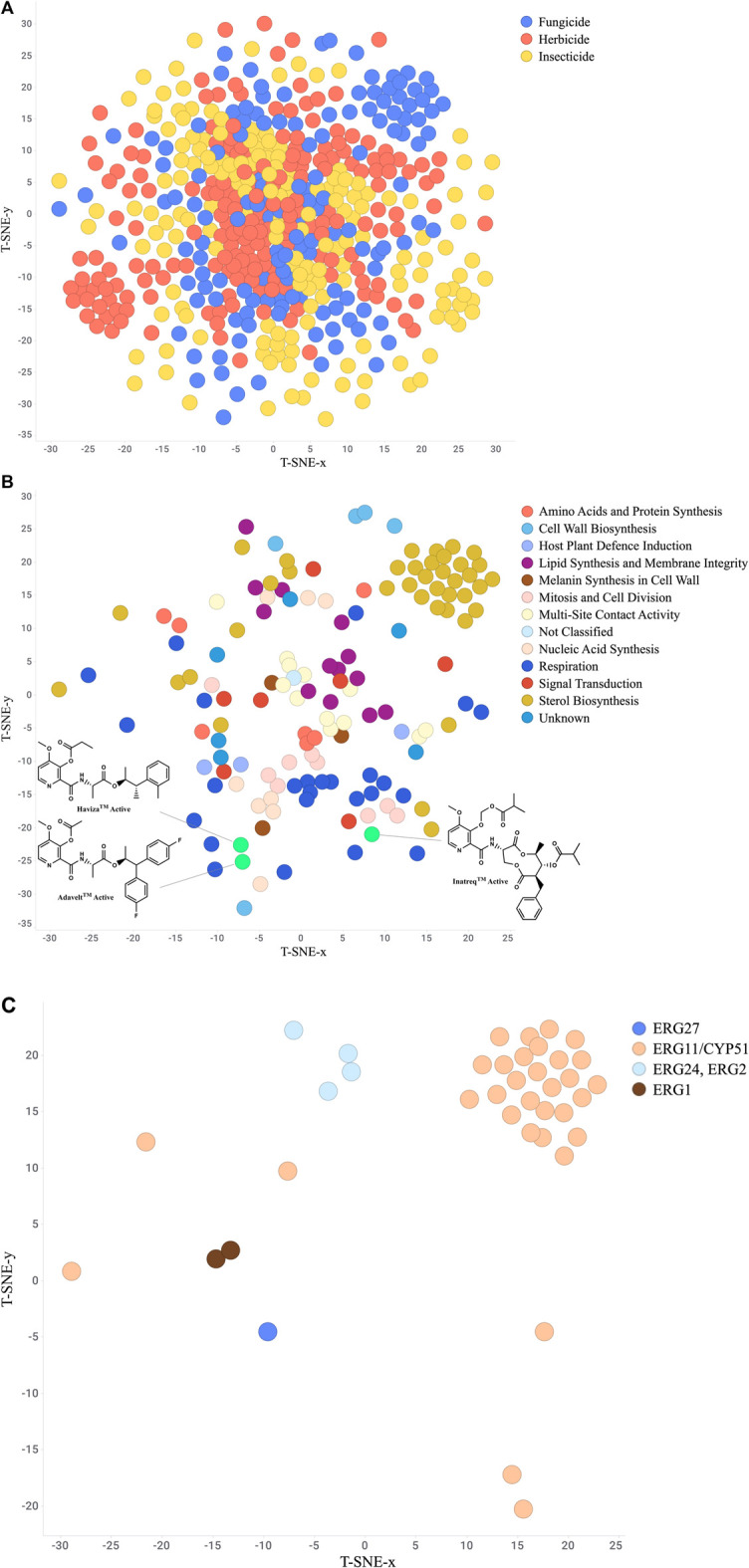
(Continued).

One valuable visualization for the researcher is a “Chemistry-Space Map”. This is often called a star-field map due to its similarity to nighttime sky. Each compound is mapped in a 2D or 3D space in such a way as to group the most similar compounds together while still showing the relationships to more dissimilar compounds. The layout is created using a set of structure descriptors and then analyzed using a numerical approach such as t-distributed stochastic neighbor embedding (t-SNE) (Karlov et al., 2019; Andronov et al., 2021), Uniform Manifold Approximation and Projection (UMAP) (McInnes et al., 2018), or Tree MAP (Probst and Reymond, 2020). These maps provide a useful structure-based organization of the project chemistry which can then be analyzed further by layering on the biological results (Janssen et al., 2019). For example, Gonçalves *et al.* (Gonçalves et al., 2021) utilized a combination of t-SNE and k-means methods to compare several hundred novel isoxazolines to commercialized isoxazoline insecticides, clearly identifying areas of novelty. Additionally, Wang et al. (Wang et al. 2022) mapped approved drugs with similar commercial herbicides to suggest isoteric replacements for novel herbicide chemotypes. Figure 8A depicts a t-SNE analysis of all FRAC, HRAC, and IRAC compounds available in ChEMBL. As this dataset does not contain the newest picolinamide fungicides Inatreq™, Adavelt™, and Haviza™, we added these structures and filtered to only fungicides to highlight this technique’s ability to qualitatively identify novel areas of chemistry (Figure 8B). The newer, non-macrocyclic picolinamides are clearly distinct from the natural product-derived macrocycle. Further filtering to only sterol biosynthesis inhibitors (Figure 8C) provides clusters at each site of action which generally overlap with chemical class.

#### 3.4.4 Metabolism

Metabolomics is a field of “omics” research that focuses on the high-throughput characterization and identification of small products of cell metabolism, called metabolites, within biological matrices (Wishart, 2008). In agriculture, the metabolite content and its alterations are related to developmental and differentiation processes, plant and fruit maturation processes, as well as resistance to external stimuli such as pathogen attacks and other environmental factors (Ibarra-Estrada et al., 2016). From an active/lead discovery standpoint, how a molecule is metabolized within a biological species influences its mode of action, bioactivity, and toxicity, among other parameters (Aliferis and Chrysayi-Tokousbalides, 2011). Compared to pharmaceutical discovery, metabolomics studies in agrochemical discovery encompass a larger and more complex set of biological systems, as pesticides are applied on and around crops. These systems include pests (insects, weeds, fungi, fish, etc.), the crops to be protected, and the surrounding environment (non-target organisms, soil, water, etc.). While in the early design phases scientists may often promote molecules with no major toxic metabolites to rapidly discard toxic molecules, or control the metabolism/activation process of the future active ingredient (Jeschke, 2016), it becomes important, as molecules progress through the lead optimization stage, to identify all metabolites for toxicity and environmental fate (U.S. EPA, 2023a). Moreover, less toxic yet non-degradable compounds are of concern too, due to bioaccumulation potential. For these reasons, a deep understanding of small molecule metabolism and environmental fate at early discovery stages would allow rapid screening of millions of compounds to prioritize the more promising ones, thus leaving more time and resources for synthesis and other phases of the DMTA cycle (Clark, 2018) or simply to accelerate the whole discovery process. Thus, over the last two decades, cheminformatics and AI-based tools have become ubiquitous in the development of cost-effective tools for automated metabolite elucidation and metabolic data interpretation.

A major bottleneck in metabolomics is the structural elucidation of small molecules detected in metabolism and environmental fate studies (Wishart, 2008). Typically, this involves the acquisition of spectra (mass or nuclear magnetic resonance (NMR)) from biological or environmental samples, followed by their processing and matching against reference spectral databases to identify the corresponding chemical structure(s) (See Table 3). Unfortunately, current libraries are neither comprehensive nor structurally diverse enough to support the retrieval of all known metabolites, which often leads to extremely low identification rates (<2%) in untargeted metabolomics experiments (da Silva et al., 2015). To address this data scarcity and many other challenges of structure elucidation, *in silico* approaches usually follow the assumption that structurally similar compounds possess similar fragmentation patterns and properties under similar conditions (Schollée et al., 2017). To assess and leverage this “quantitative structure-fragmentation relationship” (QSFR), they generally combine cheminformatics concepts such as molecular fingerprints (Rogers and Hahn, 2010), structure-based classification (Djoumbou et al., 2016; Kim et al., 2020), chemical-informed clustering (R. Ash, 2019), and structure/reaction representation languages (Daylight Chemical Information Systems, Inc, 2019a; Daylight Chemical Information Systems, Inc, 2019b) with machine learning approaches, ranging from random forest to DNNs (Liebal et al., 2020). Notable contributions to improve metabolite identification workflows include, among others, tools for spectra pre-processing (Li and Wang, 2019; Melnikov et al., 2020), the prediction of MS and NMR spectra from molecular structures (Castillo et al., 2016; Wang et al., 2021b; Hong et al., 2023), as well as the prediction of molecular structures and features from MS spectra (Wang et al., 2021c; Dührkop et al., 2021) (See Table 3). Altogether, such ML tools can be used to propose chemical structures without database search or expanding reference databases (Guijas et al., 2018; Wishart et al., 2018; Djoumbou-Feunang et al., 2019b) to boost the identification rates.

The identification of (major) metabolites and metabolic pathway, and the expansion of metabolome databases in particular, could be further facilitated by using metabolism and environmental fate prediction tools, which can suggest biologically feasible and relevant structures. Such tools are thus relevant not only in early (e.g.: molecule design, lead optimization, ADME-Tox profiling) but also in late (e.g.: metabolism and environmental fate studies, ecotoxicological risk assessment) stages of the agrochemical discovery process (Clark, 2018). Currently, most of the available software tools focus solely on human/mammalian metabolism (Litsa et al., 2020; SimulationsPlus, 2023), and environmental microbial metabolism (Wicker et al., 2016). Only a few tools (Djoumbou-Feunang et al., 2019a; QSAR Toolbox, 2023) allow comprehensive prediction for several biological systems including human hepatic and gut microbial, environmental microbial, etc. Unfortunately, most of these tools have been developed using training data mostly comprising drugs and drug-like molecules and perform less well on ag-relevant chemistries. Moreover, xenobiotics metabolism is difficult to predict, as several factors (polymorphisms, expression levels, reference data scarcity, etc.) affect the training of predictive models. Another limitation to the comprehensive prediction of metabolism and biodegradation products obtained from agrochemicals is that software tools that predict plant metabolism (Karp et al., 2019; Pathway Tools, 2023), and abiotic transformations (U.S. EPA, 2023b) of small xenobiotics are scarce at best. Furthermore, the increasing emphasis on pollinator-friendly farming implies that there is an urgency to develop and share computational tools and resources that can enable the prediction/elucidation of metabolites in pollinator species (e.g.: bees), and how metabolic alterations affect them (du Rand, 2015). These limitations must be addressed by harnessing data from publicly available regulatory reports and private data. Furthermore, scientists should leverage recent works in the area of synthetic reaction prediction, as the methodologies and algorithms could apply to metabolism prediction as well. For instance, Litsa *et al.* developed transformer-based, template-free model for mammalian drug metabolite prediction, with comparable performance to template-based models such as BioTransformer and SyGMa (Litsa et al., 2020).

The analysis and interpretation of metabolic data is another very cumbersome task in metabolomics. Such analyses are conducted to study the response of plants and target species to external stimuli, and identify metabolic/biosynthetic pathways, among other tasks. Some of the most promising approaches rely on statistical analysis, as well as substructure- and network-based methods, which are often combined with machine/deep learning. Readers are referred to publications by De Souza *et al.* (De Souza et al., 2020), Ramos *et al.* (Ramos et al., 2019), and Beniddir *et al.* (Beniddir et al., 2021) for a comprehensive review of such methods. Furthermore, several recent articles provide a comprehensive review on the applications of AI in metabolomics (Uppal et al., 2016; Heinemann, 2019; Liebal et al., 2020; Pomyen et al., 2020). For a list of software tools and resources that enhance metabolomics, readers are referred to Table 3.

## 4 Impact of cheminformatics on sustainability and environment-friendly programs

Insect population decline (Belsky and Joshi, 2019; Sánchez-Bayo and Wyckhuys, 2019) has, partly due to use of agrochemicals, led to the development of novel strategies to promote ecological-resilience and sustainable crop protection. Examples of such strategies include designing new broad-utility nitrogen stabilizers with improved safety, and herbicides with novel, un-exploited, or proven efficient mode of action for effective and durable control of driver weeds in crops. Within just a few decades, the term sustainability has gained in popularity and significance. Recent agricultural methods are far more efficient than those farmers used a few decades ago, primarily due to advancements in technology such as using big data in agricultural practices to characterize chemical toxicity and impacts on human wellbeing and ecosystem health, and advancements in plant genotyping methods and sequencing as promising tools for plant breeding and genetics research. The insights and recommendations derived from these advanced data analytics and bioinformatics tools together with the adoption of precision agriculture technologies guide us toward having safer, efficient, and more environmentally friendly alternatives.

Mathematical and *in silico* models are being used in food (from farm to fork) and agriculture sectors for sustainable and resilient systems with the general goal of providing safer food and transitioning to more sustainable farming. For instance, DynamiCrop is a plant-uptake multi-compartment mathematical model (DynamiCROP, 2023) used for the assessment of human exposure from pesticide residues in food crops. The model uses databases of reference plant dissipation half-lives of 333 pesticides in crops to estimate the amount and traces of pesticides in multiple crops and also to estimate human health impacts due to the uptake of pesticides (Peter et al., 2014). Apart from dynamic mathematical models, *in silico* models and tools in the agriculture industry are being considered inexpensive and fast alternative approaches to toxicological and ecological assessments (Supratik et al., 2017). Short-term toxicity assays such as Ames-mutagenicity and carcinogenicity (Benfenati et al., 2019), skin sensitization (Borba et al., 2021), and bee toxicity (Carnesecchi et al., 2020b) are examples of toxicity assessments that can be assisted by *in silico* models for prediction of such toxicological testing where prediction on a new pesticide candidate can be made merely by using the chemical structure.

Natural products have long been used as pesticides and have broadly served as a source of inspiration for synthetic organic fungicides, herbicides, and insecticides. Natural products are produced by biosynthetic enzymes and pathways. Cheminformatics tools can enhance structural characterization and activity specification of pesticidal natural products, and thus, make substantial contributions to the renewed field of natural product discovery (Chen and Kirchmair, 2020). During the hit-to-lead and lead optimization phases, ML approaches have been applied to natural products for predicting bioactivity and their protein targets (Olğaç et al., 2017; Cockroft et al., 2019). For example, STarFish (Cockroft et al., 2019) uses publicly available natural product databases and implements a stacked ensemble approach that combines multiple ML classification models to predict the protein target for the bioactive natural product. A recent publication uses machine learning classifiers to predict antibacterial or antifungal activity directly from natural product biosynthetic gene clusters (Walker and Clardy, 2021).

An example of commercially successful natural pesticides are Spinosyns, a large family of substances produced from fermentation of a soil inhabiting bacterium (*Saccharopolyspora spinosa*) (Kirst, 2010). Two insecticidal products have been commercialized from spinosyns: 1) Spinosad, a naturally occurring mixture of spinosyn A and spinosyn D, and 2) Spinetoram, a semi-synthetic derivative of spinosyns (Sparks et al., 2021). Spinosad received an expedited review and has been registered for integrated pest management and insect control by EPA since early 1997 (National Pesticide Infomation Center, 2023). It is valuable in control of insect pests, while minimizing the impact on beneficial insects (Sarfraz et al., 2005), and has a favorable environmental profile as it does not persist in the environment. Moreover, since Spinosad adsorbs to the soil with a higher affinity (especially in soil-clay), leaching through unsaturated soil to groundwater resources is minimized (Sparks et al., 2021). Molecular modeling using cheminformatics and AI tools has contributed, among others, to the discovery of spinosyns, and in particular, to the development of the semi-synthetic product, Spinetoram, with improved insecticidal efficacy and expanded spectra (Sparks et al., 2008; Sparks et al., 2021), which is considered a new milestone in the age of natural product-based insecticide discovery and crop protection research.

## 5 Current challenges, and future perspectives

As discussed throughout this review, cheminformatics and artificial intelligence can significantly enhance the design and development of novel and more sustainable crop protection agents. Yet, several factors still limit the wider adoption of *in silico* tools throughout the process. The scarcity of standardized, high-quality agrochemical datasets remains a challenge that hampers several processes, including but not limited to knowledge extraction (e.g.: via semantic-based querying), and predictive modeling.

A rising concern in agrochemical research is the environmental impact of pesticides. This has led to a renewed interest in natural products as pesticides. Besides the methods presented here, metagenomics-based approaches have been developed that provide a means to mine and link the metabolome and genome of species of interest. These techniques can be especially useful in the identification of natural agrochemicals and species-specific target genes. Moreover, the study of gene/protein mutations in resistant pests can be investigated to identify the underlying mechanism and suggest actions for the design of more potent agrochemicals or propose new modes of action. Validation of the proposed hypothesis can only be achieved if protein structures are available for large and diverse sets of ag-relevant species (e.g., pests, pollinators). This is, however, a bottleneck in agchem research that impedes the discovery of novel protein targets and modes of action, as well as the generation and optimization of molecules. In 2021, significant milestones were achieved in the prediction of protein structures based on sequence information with the publication of AlphaFold (Jumper et al., 2021) and RoseTTAFold (Baek et al., 2021). These predictive models could be used, along with other *in silico* solutions, to develop theoretical models and annotate protein-ligand complexes (Simonovsky and Meyers, 2020; Agaarwal et al., 2021; Hekkelman et al., 2023), which could be deposited in public databases (Varadi et al., 2022). For specific discovery projects, relevant models can then be probed using solutions described throughout the paper for hit/active identification, target selection, mode-of-action detection, and the design of *in vitro* protein assays. Furthermore, molecules selected through virtual screening or (generative) *de novo* design, can be synthesized efficiently, and tested *in vitro* to provide data for further (QSAR) analysis.

The rapid development and efficient use of cheminformatics and ML tools requires capabilities to generate, process, store, and transfer data between various environments. Moreover, developing the best tools usually requires probing larger spaces with respect to data, algorithms, and parameters. Fortunately, frameworks for distributed storage and processing (Dask, 2023; Spark, 2023), containerization (Docker, 2023), and orchestration (Kubernetes, 2023), among others, can be used for the development of scalable cheminformatics and ML solutions, and seamless integration with diverse pipelines. Ideally, deployed tools should be coupled with user interfaces providing capabilities for visualization, data manipulation, and better user experience for chemists and biologists. Moreover, efficient means for feedback loops and timely updates of software tools are needed to further engage users (Volkamer et al., 2023). Platform-as-a-service (PaaS), and Model-as-a-service (MaaS) cloud computing solutions can enhance the monitoring, use, and automated release of *in silico* solutions. However, such platforms cannot be designed and used efficiently without significant expertise and close collaborations between all parties involved (scientists and engineers). To alleviate these challenges, chemistry aware *in silico* tools such Torx^®^ (Torx Software Ltd, 2023) and LiveDesign^®^ (Schrödinger, 2023a) have been developed to help manage the complex workflows of compound synthesis, hypotheses tracking, assay cascades, computational analyses, and compound genealogy in a collaborative way.

While major developments have occurred in both experimental and predictive discovery procedures, it is clear that a paradigm shift towards a more AI-involved approach requires a gradual implementation of inter-connected automated software and hardware solutions capable of generating, prioritizing, and validating explainable hypotheses (with or without human biases) upon integrative data analysis for better decision making. This is a fairly complex task, which cannot be achieved independently, and requires cross-collaborations, not only within but between institutions. Several public private consortia have been created over the last 5 years with the aim of developing comprehensive computational discovery and synthesis platforms. Examples include the MLPDS (MLPDS, 2023), the MELLODDY (MELLODDY, 2023), and the ATOM consortia (ATOM, 2023). While these alliances have predominant membership from pharmaceutical companies, we strongly believe that the agrochemical industry would benefit from joining.
